# Increased autoreactivity and maturity of EBI2^+^ antibody-secreting cells from nasal polyps

**DOI:** 10.1172/jci.insight.177729

**Published:** 2024-09-10

**Authors:** Junqin Bai, Atsushi Kato, Kathryn E. Hulse, Joshua B. Wechsler, Vikram Gujar, Julie A. Poposki, Regan Harmon, Naruhito Iwasaki, Bao-Feng Wang, Julia H. Huang, Whitney W. Stevens, David B. Conley, Kevin C. Welch, Robert C. Kern, Anju T. Peters, Stephanie C. Eisenbarth, Robert P. Schleimer, Bruce K. Tan

**Affiliations:** 1 Department of Otolaryngology,; 2 Division of Allergy and Immunology, Department of Medicine, and; 3 Departments of Pediatrics and Medicine, Northwestern University Feinberg School of Medicine, Chicago, Illinois, USA.; 4 Department of Anatomy and Cell Biology, Oklahoma State University, Tulsa, Oklahoma, USA.; 5 Department of Otolaryngology-Head and Neck Surgery, Tongji Hospital, Wuhan, China.

**Keywords:** Immunology, Inflammation, Adaptive immunity, Immunoglobulins, Respiration

## Abstract

Elevated numbers of antibody-secreting cells (ASCs) and anti–double-stranded DNA (anti-dsDNA) antibodies are found in nasal polyp (NP) tissue. The presence of anti-dsDNA IgG in tissue prospectively predicts recurrent NP but the characteristics of the source ASCs are unknown. Here, we investigated whether NP B cells expressing the extrafollicular marker EBI2 have increased propensity for autoantibody production and evaluated the molecular characteristics of NP ASCs. NPs showed increased frequencies of anti-dsDNA IgG and total IgG ASCs compared with tonsils, with more pronounced differences among EBI2^+^ cells. In NPs, EBI2^+^ cells were frequently double negative (IgD^–^CD27^–^) and ASCs. Single-cell RNA-Seq analysis of tonsils and NPs revealed substantial differences in B lineage composition, including differences in percentages of ASCs, germinal centers, proliferative cells, and non-ASCs. NPs exhibited higher expression of specific isotypes (*IGHE*, *IGHA1*, *IGHA2*, and *IGHG4*) and mature plasma genes, including *SDC1* and *XBP1*, than tonsils. Gene Ontology biological processes indicated upregulated NF-κB and downregulated apoptosis pathways in NP ASCs. Together, these data indicate that NP EBI2^+^ ASCs secret increased total and anti-dsDNA IgG compared with those from tonsils and had molecular features of mature plasma cell differentiation.

## Abstract 2

Nasal polyps have increased EBI2+ cells that secrete anti-dsDNA- and total- IgG. Antibody secreting cells (ASCs) show distinct iso/subtype expressions, mature plasma cell features and differential activation signaling pathways from their counterparts in t

## Introduction

Chronic rhinosinusitis (CRS) affects approximately 12% of adults in the United States, where it is commonly characterized by type 2 (T2) inflammation (1). We previously described elevated levels of autoreactive antibodies against double-stranded DNA (dsDNA) and phospholipids (2–4) as well as robust antibody-mediated complement activation (5) in nasal polyp (NP) tissue compared with tissue from individuals acting as healthy controls. Anti-dsDNA IgG was separately established as a strong independent prospective risk factor for NP recurrence, equivalent to IL-5 (6). It remains unclear whether autoantibodies in NPs are synthesized locally or accumulate from plasma leak or in complex with tissue autoantigens. NPs have elevated levels of T2 cytokines that are correlated with the anti-dsDNA levels but can directly activate B lineage cells (7, 8). Autoreactive responses are also described in severe T2 asthma, suggesting that similar phenomena may occur in the unified lower airway (9).

Mechanisms of B cell activation within a secondary lymphoid organ like tonsils are well characterized (10, 11). Naive B cells encountering antigens either localize to germinal centers (GCs) or migrate to extrafollicular (EF) sites. Within the GC, activated B cells express B cell lymphoma 6 (BCL6), differentiate, and migrate into the T and B cell–rich zones (12, 13). B cells undergo tightly regulated antigen-driven activation through interaction with cognate Th and T follicular helper cells, resulting in proliferation, class-switch recombination (CSR), somatic hypermutation (SHM), and antigen-affinity selection (14). CSR alters the antibody heavy chain isotype, while SHM modifies its affinity and specificity. GC-activated B cells become memory B cells or differentiate into short-lived plasma cells (SLPCs), whose lifespan is limited by apoptosis, or long-lived plasma cells (LLPCs) that can migrate to the bone marrow, where they durably generate high-specificity antibodies. EF responses are less well characterized but known to generate polyreactive antibodies through less coordinated processes compared with the GC response (14, 15). The Epstein Barr virus–induced G protein–coupled receptor 2 (EBI2), encoded by *GPR183*, is a chemoattractant receptor, expressed on mature B cells, that directs them from GC to EF sites where they maintain EBI2 expression (16–18). Although many textbooks describe CSR and SHM as exclusive to GC reactions, these phenomena are now recognized to occur in EF-activated B cells (14, 19). The features and longevity of EF-generated antibody-secreting cells (ASCs) remain unclear, but circulating EF-activated ASCs are increasingly recognized for their pathogenic roles in autoimmune diseases (20).

Conflicting data exist on whether B cells in CSR with NPs (CRSwNP) are activated via GC or EF responses. Some studies describe GC-like follicular structures, high endothelial venules, and T follicular helper cells in NPs (21, 22) and interpret expression of germline gene transcripts and presence of switch circles, indicating tissue-localized CSR (7), as evidence of a localized GC reaction. In contrast, our histologic study found poorly organized lymphoid clusters in CRSwNP, and these were not elevated in density compared with control tissue. We also observed increased EBI2 expression on NP ASCs, suggesting EF activation (23). Similarly, Corrado et al., utilizing lineage tracing of the heavy chain, concluded that the EF response generated IgE ASCs in NPs (24).

In this study, we focused on identifying whether the aforementioned anti-dsDNA IgG antibodies are secreted by tissue-resident B cells and characterizing molecular features of these ASCs. Flow cytometry and ELISpot results provided compelling evidence that NP ASCs, especially those expressing EBI2, were profoundly dsDNA autoreactive. Single-cell RNA-Seq (scRNA-Seq) analysis of NP ASCs compared with tonsil ASCs uncovered distinct transcriptomic profiles and signaling pathways utilized by NP ASCs. These findings demonstrate that NP ASCs are dsDNA autoreactive and provide insight into mechanisms by which plasma cells may promote chronic and refractory inflammation in CRSwNP.

## Results

### Phenotyping of B cell subsets in NPs compared with tonsils.

**Figure 1:**
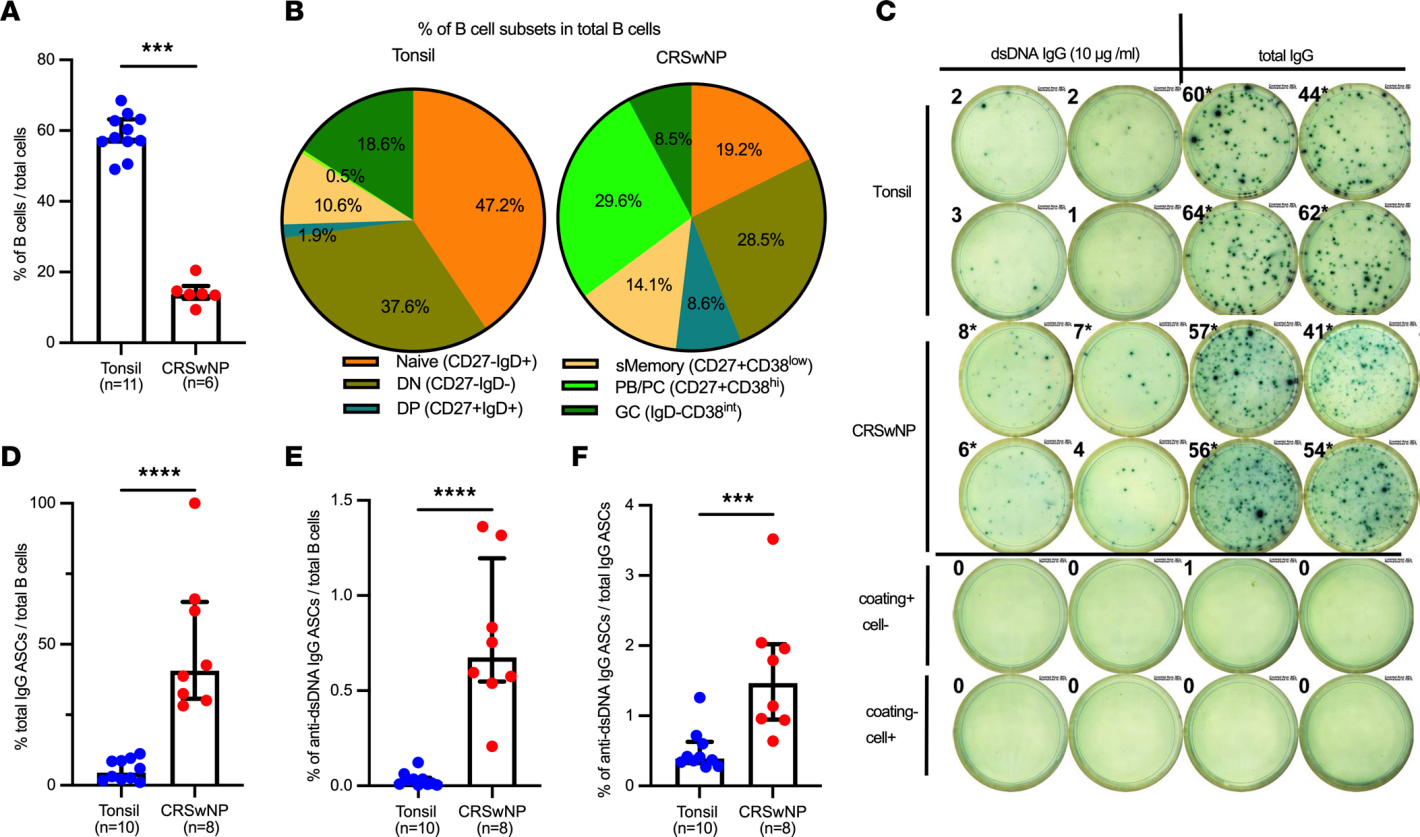
NP tissue contained increased anti-dsDNA IgG and total IgG-secreting cells. (**A**) Bar plot showing the frequency of B cells in all cells in tonsils (*n* = 11) and NPs (*n* = 6). (**B**) Pie charts showing the frequency of B subsets in B cells derived from tonsils (*n* = 11) and NPs (*n* = 6) enumerated by flow cytometry. (**C**) Frequencies of dsDNA-specific (10 μg/mL) and total IgG-expressing ASCs were quantified by ELISpot. (**D**–**F**) The frequencies of ASCs were compared between tonsils (*n* = 10) and NPs (*n* = 8): (**D**) IgG ASCs per B cell, (**E**) anti-dsDNA IgG ASCs per B cell, and (**F**) anti-dsDNA IgG ASCs per IgG ASC. The Mann-Whitney *U* test was used for the comparisons. ***P <.001, ****P < .001. Median with IQR is indicated.

As an extension of our previous studies (23), we examined B cell heterogeneity in NPs and tonsils using flow cytometry (gating strategy in Supplemental Figure 1; supplemental material available online with this article; https://doi.org/10.1172/jci.insight.177729DS1). We verified that B lineage cells (CD3^–^CD19^+^) per live cell were higher in tonsils (58.9%, *n* = 11) than in NPs (14.2%, *n* = 6) (*P* < 0.001) (Figure 1A). The most expanded subsets in NPs compared with tonsils were plasmablasts (PB) and plasma cells (PCs) (CD27^+^IgD^–^CD38^hi^) (29.6% vs. 0.5%, 59-fold, *P* < 0.001) (Figure 1B and Supplemental Figure 1B). NPs had significantly less frequent naive (CD27^–^IgD^+^) (19.2% vs 47.2%, *P* < 0.01) and IgD^–^CD38^int^ B cells (8.5 % vs 18.6%, *P* < 0.05) than tonsils. In prior publications, including our own, IgD^–^CD38^int^ cells were described as a GC phenotype (23), but in the absence of specific GC markers such as *BCL6*, this is likely not definitive. Notably, and previously overlooked, in both NPs and tonsils, double negative (DN) cells (IgD^–^CD27^–^) (28.5% and 37.6%, respectively) were very common. DN cells, first described in EF regions of tonsils, are also found in the blood of patients with systemic lupus erythematosus and represent activated precursors of autoreactive ASCs (25). The DP (CD27^+^IgD^+^) cells were also more frequent in NPs than tonsils (8.6% vs 1.9%, respectively) (Supplemental Figure 1B).

### NPs contained increased anti-dsDNA IgG and total IgG ASCs.

We next quantified ASCs by ELISpot assays, measuring total IgG and anti-dsDNA–specific IgG ASCs (Figure 1C, Supplemental Figures 2 and 3, Supplemental Table 2). While tonsils had a higher number of B cells, the frequency of total IgG ASCs per B cell was 10-fold lower in tonsils compared with NPs (4.5% vs 40.7%, *P* < 0.0001) (Figure 1D). In NPs, the frequency of dsDNA IgG ASCs normalized per B cell was 34-fold higher than in tonsils (0.68% vs 0.02%, *P* < 0.0001) (Figure 1E). The frequency of dsDNA autoreactive ASCs per total IgG ASC was also higher in NPs compared with that in tonsils (1.5% vs 0.4%, *P* < 0.001) (Figure 1F), indicating that NP ASCs were more likely to secrete IgG and be autoreactive.

### IgG ASCs and anti-dsDNA IgG ASCs were enriched in sorted EBI2^+^ cells.

**Figure 2:**
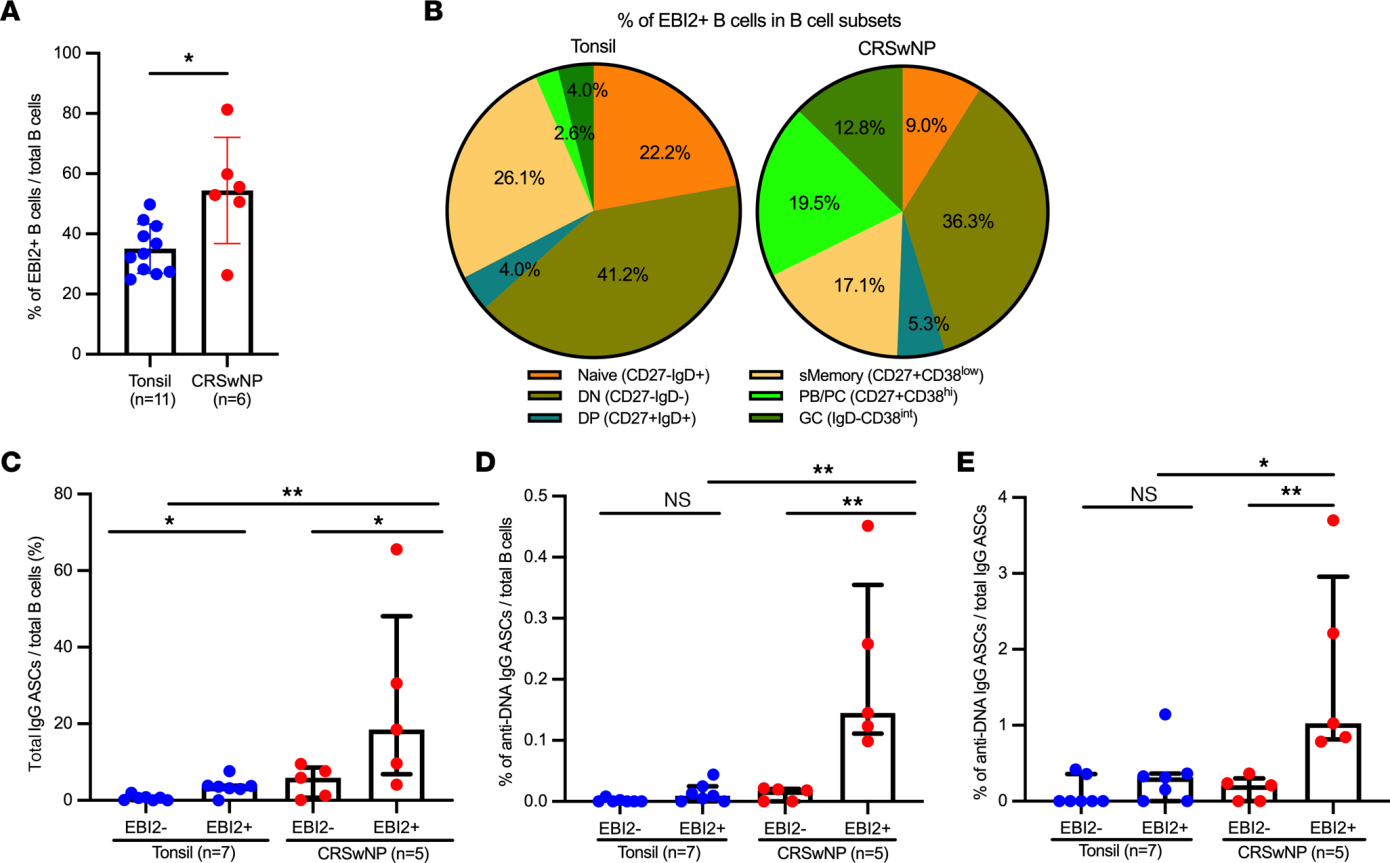
Frequencies of IgG ASCs and anti-dsDNA–specific IgG ASCs are enriched in sorted EBI2^+^ cells. (**A**) Bar plot showing the frequency of EBI2^+^ cells in B cells in tonsils and NPs. (**B**) Pie charts showing the frequency of EBI2^+^ cells in B cell subsets derived from tonsils (*n* = 11) and NPs (*n* = 6) determined using flow cytometry. sMemory, class-switched memory. (**C**–**E**) Comparisons of total and dsDNA-specific ASCs in B cells from tonsils (*n* = 7) and NPs (*n* = 5): (**C**) IgG ASCs in total B cells, (**D**) anti-dsDNA IgG ASCs in total B cells, and (**E**) anti-dsDNA IgG ASCs in total IgG ASCs determined by ELISpot. The Mann-Whitney *U* test was used for the 2-group comparisons. The Kruskal-Wallis test with Dunn’s correction was used for the multiple group comparisons. If *P* > 0.05, the result is not significant (ns). *P < 0.05, **P < 0.01. Median with IQR is indicated.

Using flow cytometry (26), we found that EBI2^+^ B cells were enriched in NPs (*n* = 6) compared with those tonsils (*n* = 11) (*P* < 0.05) (Figure 2A and Supplemental Figure 1). Of these EBI2^+^ B cells, the DN phenotype was prevalent in both NPs and tonsils (36.3% and 41.2%, respectively). Additionally, a marked proportion of EBI2^+^ cells comprised class-switched memory (17.1% and 26.1%) and naive cells (9.0% and 22.2%) in NPs and tonsils, respectively. Importantly, NPs had a higher frequency of EBI2^+^ PBs/PCs than tonsils (19.5% vs 2.6%, *P* < 0.001) (Figure 2B and Supplemental Figure 1D).

ELISpot analysis of CD3^–^CD19^+^ B cells flow sorted using EBI2 expression revealed a 3-fold higher frequency of IgG ASCs per B cell in EBI2^+^ cells than the EBI2^–^ B cells in both NPs and tonsils (both *P* < 0.05) (Figure 2C). Furthermore, NP EBI2^+^ B cells exhibited higher frequency of IgG ASCs than tonsil EBI2^+^ cells per total B cell (*P* < 0.01). Autoreactive anti-dsDNA IgG ASCs were similarly more frequent in the EBI2^+^ cells than EBI2^–^ B cells in both NPs and tonsils (Figure 2D). Strikingly, these NP EBI2^+^ cells were 15-fold more dsDNA autoreactive than tonsil EBI2^+^ cells (*P* < 0.01). Accordingly, we found that the frequency of anti-dsDNA IgG ASCs per IgG ASC was higher in NP EBI2^+^ cells than in tonsil EBI2^+^ cells (3-fold, *P* < 0.05) (Figure 2E). From these results, we inferred that the EBI2^+^ B cells, particularly from NPs, were frequently ASCs producing both total and dsDNA-specific IgG.

### scRNA-Seq identified transcriptionally distinct B cell subsets.

Given the disproportionate dsDNA autoreactivity of NP ASCs and the prognostic importance of anti-dsDNA IgG in CRSwNP, we aimed to evaluate the transcriptomes of NP ASCs using scRNA-Seq analysis. Since previous unenriched scRNA-Seq studies in CRSwNP failed to distinguish ASCs from other B lineage cells, we performed a negative selection prior to performing scRNA-Seq (27–29).

**Figure 3:**
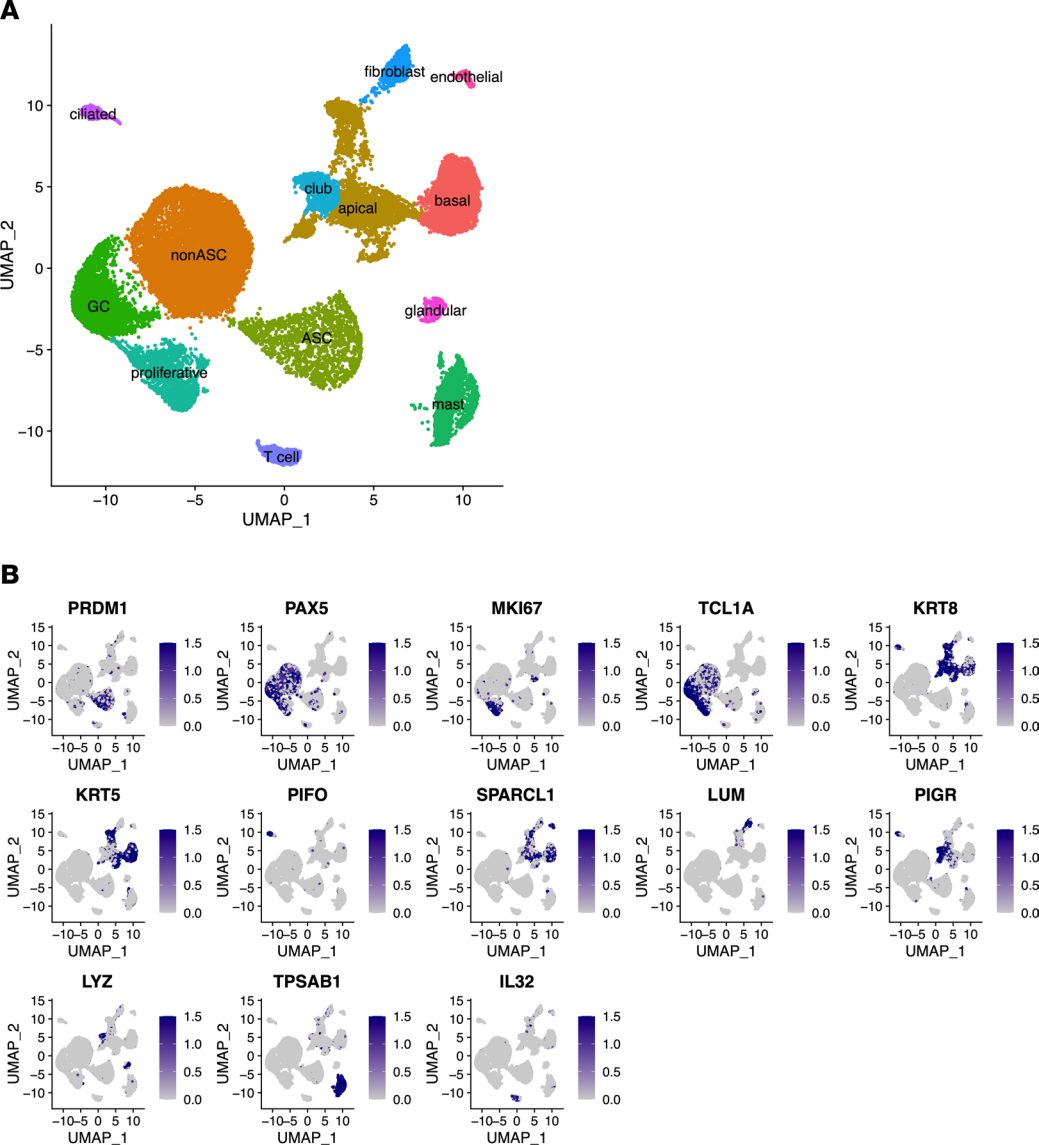
Characterization of B cell subsets using scRNA-Seq and CITE-Seq analyses. (**A**) Single-cell analysis of cells from patients with tonsil (*n* = 5) and CRSwNP (*n* = 5). Two-dimensional UMAP plots of 28,192 cells. (**B**) Feature plots of characteristic genes in different cell types.

We identified 28,192 cells from NP and tonsil extracts from a total of 10 donors (*n* = 5 each). Based upon expression of characteristic genes, we identified 13 distinguishable clusters visualized in a feature plot (Figure 3A), of which 4 were B cell lineage (Supplemental Results). Tissue type–consistent donor contributions were visualized with the uniform manifold approximation and projection (UMAP) algorithm (Figure 3B).

**Figure 4:**
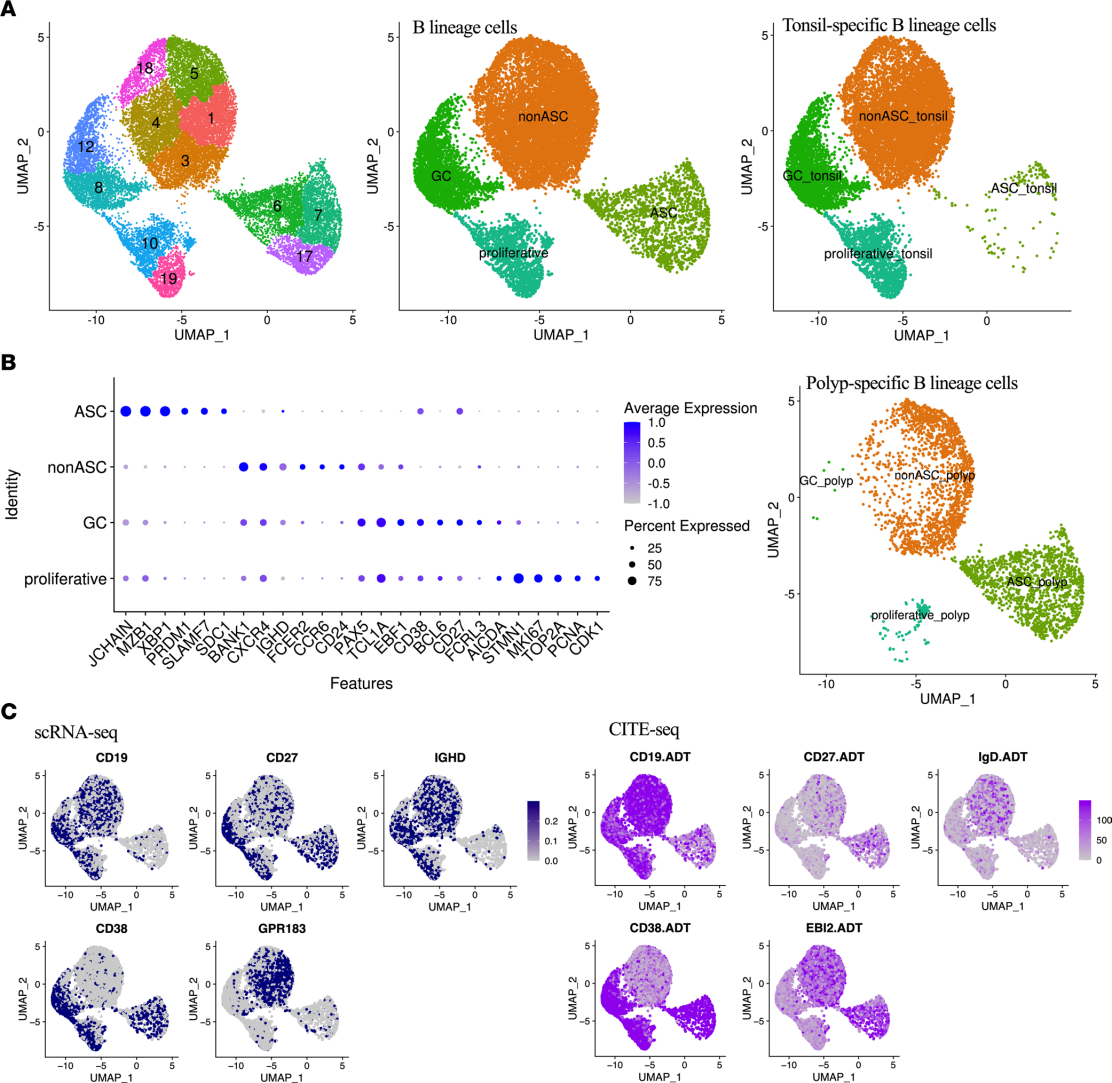
Two-dimensional UMAP plots of B lineage cells. (**A**) UMAP plots of B lineage cells separated by tissue type. (**B**) Dot plot of marker genes of B lineage cells: expression levels across B cell subsets. (**C**) Discrete ASC signatures by scRNA-Seq (left) and CITE-Seq (right) analysis.

Characteristic genes for the B cell clusters (30–32) were specifically analyzed to identify ASC (*PRDM1*, *XBP1*, *JCHAIN*, *SLAMF7*), non-ASC (*CCR6*, *BANK1*, *FCER2*), GC (*BCL6*, *AICDA*, *TCL1A*), and proliferative (*MKI67*, *TOP2A*, *PCNA*, *CDK1*) subsets (Figure 4, A and B). The non-ASCs, a likely heterogeneous set of B cells, comprising naive, memory, and DN B cells found in flow cytometry, could not be further resolved transcriptomically. Nonetheless, 44.1% of NP B cells were ASCs compared with only 1.2% of tonsil B cells (37-fold increase in NPs). NPs also had 52.7% non-ASCs, 0.2% GCs, and 3% proliferative cells, whereas tonsils had 63.1% non-ASCs, 23.1% GCs, and 12.6% proliferative cells, which confirmed flow cytometric findings of the profound expansion of ASCs and absence of the GC phenotype in NPs (Supplemental Figure 5).

We validated transcriptomically mapped B lineage cells using cellular indexing of transcriptomes and epitopes by sequencing (CITE-Seq) analysis with expression of 5 surface proteins (CD19.ADT, CD27.ADT, CD38.ADT, IgD.ADT, and EBI2.ADT) (Figure 4C). The analysis confirmed consistency between surface protein expression and transcriptomically defined clusters, i.e., *CD19*/CD19.ADT concordantly labeled most non-ASCs, GCs, and proliferating B cells and *IGHD/*IgD.ADT labeled most non-ASCs. Similarly, *CD38*/CD38.ADT labeled ASC, proliferating B cell, and GC subsets, although CD38.ADT expression levels were higher in the ASCs. We did find some discrepancies between the transcriptomic and proteomic assessments of expression of *CD27, IGHD,* and *GPR183/*EBI2. *CD27* expression was observed in all 4 B lineage clusters, but CD27.ADT was mostly expressed on ASCs not on GCs or proliferative clusters, suggesting that CD27.ADT had consistency with flow cytometry–based definitions of ASCs. *IGHD* was expressed on GCs and proliferative B cells, while IgD.ADT was not expressed on them. In addition, EBI2.ADT-labeled ASCs were found at a high frequency despite the apparent lack of *GPR183* expression on ASCs (Figure 4C). Comparing our CITE-Seq results with flow cytometry data, we are confident of the validity of the characterization of ASCs and recapitulated the population identified using flow cytometry (CD27^+^CD38^hi^).

### Comparison of NP- and tonsil-derived ASCs demonstrated dramatic differences in isotype utilization, B cell differentiation, and cell signaling.

**Figure 5:**
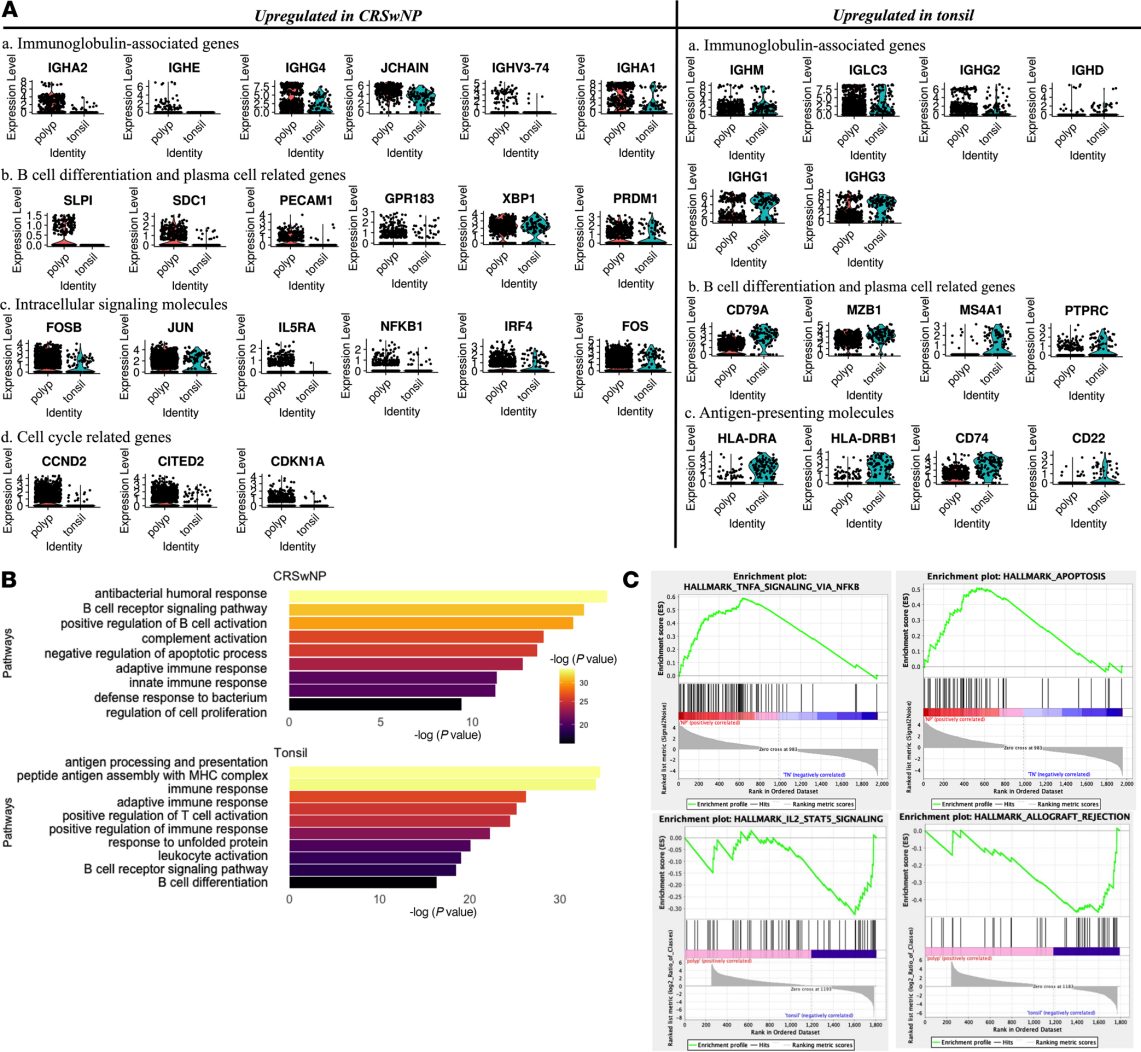
Comparison of NP- and tonsil-derived ASCs. (**A**) Violin plots of genes upregulated in NP- and tonsil-derived ASCs. (**B**) GO biological process performed on most variable genes in ASCs derived from NPs and tonsils. Wilcoxon’s test was used for gene comparisons. (**C**) GSEA was performed on the top 2,000 most variable features in ASCs derived from NPs and tonsils. NF-κB ranked as the most-enriched signaling pathway in NPs.

Next, to gain molecular mechanisms that drive the increased antibody and autoantibody expression found in NP ASCs, we compared transcriptomes of the 1,446 polyp-derived ASCs with those of 147 tonsil-derived ASCs. Differentially expressed genes (>1.5-fold) could broadly be segregated into 4 categories. (a) Immunoglobulin iso/subtype heavy chains and related genes, especially *IGHA2*, *IGHE*, *IGHG4*, *JCHAIN*, *IGHV3-74*, and *IGHA1*, were dramatically upregulated in NPs (14.0-, 10.3-, 8.3-, 4.3-, 4.3-, and 3.9-fold, respectively; all *P* < 0.0001), while *IGHM*, *IGLC3*, *IGHG2*, *IGHD*, *IGHG1*, and *IGHG3* were upregulated in tonsils (4.8-, 3.9-, 2.8-, 1.7-, 1.7-, and 1.7-fold, respectively, all *P* < 0.0001), indicating differential CSR preferences in NP- and tonsil-derived ASCs (Figure 5A and Supplemental Table 1). Interestingly, a specific IGHV region, *IGHV3-74,* was found to be increased in NPs compared with tonsils despite the 3′ sequencing bias (Figure 5A and Supplemental Figure 6). This IgHV region had previously been identified as the most commonly used IGHV by dsDNA-specific autoantibodies (33, 34). (b) PC-related genes (35, 36), including secretory leukocyte peptidase inhibitor (*SLPI*), adhesion molecule syndecan-1 (*SDC1*/CD138), platelet endothelial cell adhesion molecule 1 (*PECAM1*/CD31), *XBP1*, and *PRDM1*, were upregulated in NPs (2.4-, 2.1-, 1.8-, 1.7-, and 1.7-fold, respectively; all *P* < 0.0001), whereas B lineage markers, like *CD79A*, *MZB1*, *MS4A1*/CD20, and *PTPRC/*CD45, were upregulated in tonsils. We also noted that *GPR183/*EBI2 was 1.8-fold (*P* < 0.0001) upregulated in NP ASCs compared with tonsil ASCs. (c) Intracellular signaling molecules, including *FOSB*, *JUN*, *IL5RA*, *NFKB1*, *IRF4*, and *FOS*, were upregulated in NPs (2.1-, 1.6-, 1.6-, 1.5-, 1.5-, and 1.5-fold, respectively; all *P* < 0.0001) and (d) cell cycle–arrest genes, like *CCND2* (cyclin D2), *CITED2*, and *CDKN1A* (4.6-, 2.2-, and 1.6-fold, respectively; all *P* < 0.0001), whereas antigen-presenting molecules, including MHC class II (*HLA-DR*), *CD74*, and *CD22* were upregulated in tonsils (Figure 5A and Supplemental Table 1).

To understand the cellular pathways distinguishing ASCs in NPs and tonsils, genes showing a log fold change of more than 1 were analyzed using GO analysis. The top pathways identified were antibacterial humoral response, B cell receptor signaling pathway, positive regulation of B cell activation, complement activation pathway, and negative regulation of the apoptotic process (Figure 5B). Upregulated genes involved in the negative regulation of apoptosis included *CCND2*, *CITED2*, and *PIM2*. Gene set enrichment analysis (GSEA) was performed and revealed that NF-κB signaling and negative regulation of apoptosis notably upregulated in NP ASCs (*NFKB1*, *RELA*, *JUN*, and *FOS*) (Figure 5C) as well as hypoxia pathways (data not shown). NF-κB pathway activation may drive the antiapoptotic mechanisms, given that it blocks apoptosis in ASCs (37, 38). Additionally, tissue inflammation in CRS may result in a hypoxic condition that is known to favor human PC differentiation (39). In contrast, IL-2 and allograft rejection signaling pathways were upregulated in tonsils. The results indicate that ASC activation in CRSwNP involves atypical mechanisms and highlights potential targets for inhibiting their activation.

## Discussion

B cells play important roles in inflammatory and autoimmune conditions and are the source of dsDNA IgG that is a biomarker for polyp recurrence after surgery in CRSwNP. We discovered that ASCs are highly expanded in NPs (59-fold by flow cytometry and 37-fold by scRNA-Seq) compared with a mucosa-associated lymphoid structure like the tonsil. IgG ASCs in NP were also significantly more autoreactive to dsDNA, especially when they expressed EBI2^+^, an oxysterol receptor that drives extrafollicular migration. Besides ASCs, DN (IgD^–^CD27^–^) cells, an extrafollicular activated subset of B cells, were also common in NPs, and, correspondingly, NPs had substantially fewer naive and GC cells compared with tonsils. Transcriptomic and proteomic profiling of ASCs revealed distinct immunoglobulin isotypes, IGHV selection, B cell differentiation, signaling molecules, and cell cycle–related genes in NPs compared with tonsils. Together, these results suggest extrafollicular B cell activation mechanisms may drive dsDNA autoreactive ASCs in NP. Notably, NP ASCs also expressed higher levels of PC markers, like *SDC1*, *XBP1*, and *IRF4*, and decreased expression of B cell identity markers and antigen-presenting molecules, like *MS4A1*, *HLA-DR*, *CD74*, and *CD79A*, compared with those from tonsils, indicating increased maturity. NP ASCs also expressed significantly higher *IL5RA* and genes known to be activated by IL-5–stimulated murine B cells and utilized *IGHV3-74*, a heavy chain region with known dsDNA reactivity, implicating alternate mechanisms of activation in NP ASCs. We interpret these findings to indicate that the prognostically important dsDNA IgG antibodies are secreted directly by tissue-resident, IL-5–dependent mature ASCs.

The nature of B cell activation has important implications on memory, antigen specificity, autoreactivity, and longevity of the resultant PC. Using tonsils to clearly identify GC cells, we found that NPs had few GC cells detected using a permissive (CD19^+^IgD^–^CD38^int^) flow phenotype and nearly none using scRNA-Seq using more GC-specific markers (*BCL6*/*AICDA*). These findings mirror those of Corrado et al., using histologic and transcriptomic approaches in CRSwNP (24), but contrast with the findings of Lau et al., who demonstrated tertiary lymphoid structure and presence of high endothelial venules in NPs (22). We also found that NP ASCs more frequently expressed *GPR183/*EBI2, which drives B cell migration to extrafollicular locales (16), and EBI2^+^ ASCs were more dsDNA autoreactive by ELISpot. While EBI2 expression is well characterized on Epstein Barr virus–infected B cells, the mechanisms controlling its physiologic expression remain unclear. EBI2 mediates B cell migration within GCs through its oxysterol ligand 7α,25-dihydroxycholesterol (16, 40, 41), and in a GC, its expression peaks in mature B cells, potentially guided by NF-κB signaling that we observed was upregulated in our NP ASCs (42). Furthermore, DN cells, recently demonstrated to be central to extrafollicular and autoimmune responses (25, 43), were the most common B cell phenotype in NPs. Together, these results suggest that autoreactive ASCs in NPs are likely to have EF origins. DN cells are also expanded in the airways of asthmatics with eosinophilia and autoreactivity (44, 45), suggesting that EF activation of B cells may play an important role in the pathogenesis of autoreactivity in T2 airway diseases like CRSwNP and asthma.

Compared with ASCs in tonsils, NP ASCs utilized significantly different isotypes/subtypes by scRNA-Seq; *IGHA1*, *IGHA2*, and *JCHAIN*, required for transmucosal export of IgA and IgM, were highly expressed by NP ASCs, suggesting that these tissues compartmentalized ASCs that influence mucosal immunity. Although not studied in this paper, we previously found elevated anti-dsDNA IgA in NPs (2). J-chain linked dimeric IgA also potently stimulates eosinophil degranulation that is commonly observed in tissue and nasal secretions of patients with CRSwNP (46). We further found significantly increased utilization of *IGHG4* and *IGHE* in NPs, suggesting the elevated IgG4 and IgE levels in NPs were locally secreted, whereas IgG1–3 were more commonly utilized by tonsil ASCs. These differences between NP- and tonsil-derived ASCs also align with reports that IgE and IgG4 levels are higher in NPs from patients with aspirin-exacerbated respiratory disease than in patients with CRSwNP (47). Increased IgG4^+^ PCs are also reported to correlate with the severity of eosinophilic CRS and postsurgical outcomes (48). When considering IgG autoreactivity to DNA, these findings do raise the possibility that autoantibodies in CRSwNP may disproportionately utilize the IgG4 subtype. Recent research suggests that IgG4 autoantibody subtyping correlates better with disease severity in autoimmune epithelial barrier conditions, like pemphigus vulgaris and ANCA-associated vasculitis, indicating a need to investigate this aspect in CRSwNP (49).

In addition to iso/subtype skewing, scRNA-Seq enabled us to complete an unprecedented detailed molecular analysis of ASCs in CRSwNP. Although the identified ASCs could represent PBs and PCs, they significantly upregulated *PRDM1*/BLIMP1, *XBP1*, and *IRF4* and cell cycle arrest genes that are necessary for PC development and maintenance (50, 51). Moreover, NP ASCs upregulated *SDC1*/CD138, *SLPI*, and *PECAM1*/CD31 and downregulated B cell identity genes, like *MS4A1/*CD20, *CD79A*, and *HLA-DR*, compared with those in tonsils. These upregulated features are reported to distinguish LLPCs from SLPCs in long-term cultures and bone marrow phenotyping (35, 36, 38, 52), suggesting that NP ASCs, including those with autoreactivity, may be programmed for persistence in tissue. The results of these comparisons contrast with the findings of Ramonell et al., who compared IgE ASCs with those expressing other isotypes in allergic fungal sinusitis, a specific phenotype of CRSwNP associated with fungal responses (53). They found that IgE ASCs were more immature than other class-switched ASCs but did not compare them to lymphoid-generated ASCs described in our study. Our studies further found that upregulated chemokine receptors (*CCR10* and *CCR2*) were identified in NP ASCs, potentially assisting in tissue-specific localization of these cells, similar to mucosal PCs (54). A separate study reported higher *BCL2* expression in PCs and interpreted this as evidence for LLPCs in CRSwNP (38). However, we did not observe such a difference between NP- and tonsil-derived ASCs. Studying the mechanisms for PC differentiation and maturation is challenging because survival of in vitro–cultured PCs for more than a few days requires specialized feeder cells or stimulating cytokines (31, 55, 56).

Our scRNA-Seq analysis revealed increased expression of AP-1 pathway transcription factors (*FOS*, *FOSB*, *JUN*) and NF-κB–associated (*NFKB1*, *RELA*), JAK/STAT pathway–associated (*SOCS3*), and cell cycle–related (*CCND2*, *CITED2*) genes. The NF-κB signaling pathway has been reported to be associated with IgG ASCs (32). Although the underlying mechanism for simultaneous activation of these pathways remains uncertain, it may involve IL5RA activation. IL5RA, targeted by benralizumab in clinical trials for CRSwNP and approved for asthma, is known for its role in eosinophil maturation, but its expression on human B cells is not well established (47, 57). We found exclusive expression of *IL5RA* in NP ASCs compared with tonsils, in line with a similar report on *IL5RA* overexpression in aspirin-exacerbated respiratory disease B cells (47). Murine IL5RA could drive secretory IgA production and PC differentiation in B1 cells (58). Another study transcriptomically demonstrated that IL-5 and CD38-stimulated murine B cells activated NF-κB, JAK/STAT, and cell cycle target genes (59), similar to our findings in upregulated genes in NP ASCs. These findings provide compelling evidence that IL-5 may play a role in human mucosal ASC activation.

We are aware of the limitations of our approach. We initially hypothesized that the EF activated ASCs were PBs, and CD138 was not utilized in flow cytometry to distinguish PBs from PCs. Similarly, we also could not resolve naive, DN, and memory B cells from the non-ASC cluster in scRNA-Seq despite use of CITE-Seq. Sequencing was 3′ biased, which precludes utilization of heavy chain lineage tracing of ASCs to identify their precursors. Anti-dsDNA–specific ASCs were not separated using scRNA-Seq, because methods for identifying antigen-specific PCs are still being developed. In our study, there were also marked age differences in populations of patients who underwent tonsillectomies compared with those undergoing endoscopic sinus surgery. It is possible that the increased DN B cells may partially reflect increased age of the patients with CRSwNP, as these cell types do increase in elderly populations (60). Nonetheless, our study that demonstrates tissue-localized dsDNA autoreactive ASCs are expanded in CRSwNP and elucidates mechanisms of ASC activation relevant to severe T2 airway conditions including asthma.

In conclusion, this study provides evidence for extrafollicular activation and dsDNA autoreactivity of human mucosal ASCs in CRSwNP. These ASCs are more mature, utilize different iso/subtypes and IgHV regions with known dsDNA reactivity, and exhibit differential cell signaling mechanisms than those found in tonsils, which may confer the ability to durably alter the cellular, humoral, and immune environment of the airway in these patients.

## Methods

### Sex as a biological variable

Our study examined male and female patients, and the male-to-female ratio was 14:4 in patients with CRSwNP (*n* = 18) and 9:16 (*n* = 25) in patients with tonsillitis (Table 1).

### Participants

This study recruited patients with CRSwNP (*n* = 18) and patients undergoing tonsillectomy (*n* = 25) between 2019 and 2022. The median ages of patients with CRSwNP and tonsillitis were 49.5 ± 12 years and 28 ± 10.8 years (*P* = 0.15 with Mann Whitney test), respectively (Table 1). Characteristics of participants are shown in Table 1. Additional information is provided in the Supplemental Methods.

### Flow cytometry and sorting

Single-cell suspensions were made from tonsils and NP tissue followed by cell staining to identify B cell subsets using the protocol described previously (23) and in the Supplemental Methods. All analyses were performed with FlowJo (BD Biosciences). B cell subsets (CD19^+^CD3^–^EBI2^+^ and CD19^+^CD3^–^EBI2^–^ cells) were sorted with a FACSAria II at the Flow Cytometry Core Facility.

### ELISpot

To measure the number of total IgG ASCs and the dsDNA-specific IgG ASCs via ELISpot, titrations were performed to determine suitable plating density on precoated wells with anti-IgG (detecting total IgG ASCs) and 10 μg/mL calf thymus dsDNA (detecting dsDNA-specific ASCs; Sigma-Aldrich) with validation using 3H9 dsDNA-specific antibody-transfected cell lines (see Supplemental Methods). Tissue-derived single-cell preparations were then plated and quantitated according to the manufacturer’s instructions.

### 10x Genomics scRNA-Seq and CITE-Seq

#### scRNA-Seq.

Single-cell suspensions from tissue were used, followed by B cell enrichment using human pan-B negative selection (STEMCELL). The scRNA-Seq 3′ reagent kit (10x Genomics) was used to prepare transcriptomic libraries.

#### CITE-Seq.

CITE-Seq involves the use of antibody-derived tags (ADT) conjugated to oligonucleotides to label surface proteins for detection in addition to the scRNA-Seq pipeline. Cells were labeled with TotalSeqB antibodies (Biolegend): CD19.ADT, CD27.ADT, CD38.ADT, IgD.ADT, and EBI2.ADT.

Both mRNA and ADT libraries were analyzed using R with Seurat toolkit packages (61). Methods used for quantitation and processing of RNA and ADT levels are detailed in the Supplemental Methods (Supplemental Figure 4).

### GSEA

Differentially expressed genes were assigned biological characteristics using the Gene Ontology (GO) database on Enrichr (https://maayanlab.cloud/Enrichr/) (Avi Ma’ayan laboratory, Icahn School of Medicine at Mount Sinai, New York, USA) (62) to gain further insight into their biological process. Additionally, GSEA (63) assessed the enrichment of differentially expressed gene sets to aid our understanding of their related functional pathways across groups.

### Statistics

Data were analyzed using the Mann-Whitney *U* test for binary comparisons and the Kruskal-Wallis test with Dunn’s correction for comparisons with more than 2 groups, using GraphPad Prism (version 9). A *P* value of less than 0.05 was considered significant. Violin plots were generated for genes when more than 25% of the cells in indicated groups had non-zero measurements using default code. Enrichment for genes within clusters and different tissues was determined by Wilcoxon’s test.

### Study approval

The study was approved by Northwestern University Feinberg School of Medicine Institutional Review Board. Recruited patients provided written informed consent to tissue collections during surgery for CRSwNP or tonsillectomy.

### Data availability

Single-cell RNA-Seq data are available at NCBI GEO with accession number GSE270953. The code used for data analysis can be provided upon request.

## Author contributions

JB and BKT contributed to the data acquisition, data analysis, and interpretation of the data. AK, JBW, and RPS contributed to the data analysis and interpretation. KEH, WWS, and SCE contributed to scientific advice. VG, JAP, RH, NI, BFW, JHH, DBC, KCW, RCK, and ATP contributed to patient recruitment and suggestions. All authors provided critical review of the manuscript and approved the final version for publication.

## Supplementary Material

Supplemental data

Supporting data values

## Figures and Tables

**Table 1:**
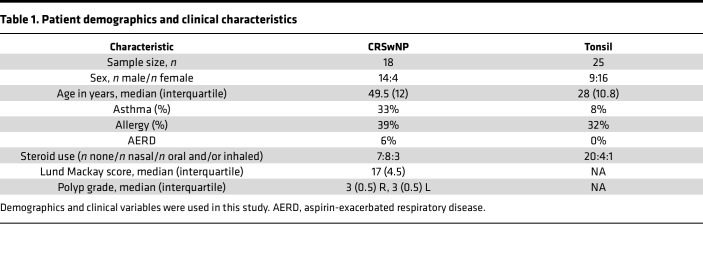
Patient demographics and clinical characteristics

## Version 1 09/10/2024

Electronic publication
