# Supplementary material for: Increased autoreactivity and maturity of EBI2+ antibody-secreting cells from nasal polyps
Source: JCI Insight. 2024 Sep 10;9(17):e177729. doi: 10.1172/jci.insight.177729 (PMC11385095; doi:10.1172/jci.insight.177729)
Supplement: Supplemental data [file jciinsight-9-177729-s217.pdf]

Online Supplement

Increased Autoreactivity and Maturity of EBI2+ Antibody-Secreting Cells from Nasal Polyps

AUTHORS

Junqin Bai, PhD, MS[1], Atsushi Kato, PhD[1,2], Kathryn E. Hulse, PhD[2], Joshua B. Wechsler, MD[3], Vikram Gujar, PhD[4], Julie A. Poposki[2], Regan Harmon[1], Naruhito Iwasaki, MD, PhD[2], Bao-Feng Wang MD, PhD[2,5], Julia H. Huang[1], Whitney W. Stevens, MD, PhD[1,2], David B. Conley, MD[1], Kevin C. Welch, MD[1], Robert C. Kern, MD[1], Anju T. Peters, MD, MS[1,2]; Stephanie C. Eisenbarth, MD. PhD[2], Robert P. Schleimer, PhD[1,2], Bruce K. Tan, MD, MS[1,2]

METHODS

Participants

Patients met the criteria for CRS defined by the American Academy of Otolaryngology–Head and Neck Surgery Chronic Rhinosinusitis Task Force (1). Recruited patients were between the ages of 19 and 76 years consented to tissue collections. Exclusion criteria for this study included patients with pregnancy, immunodeficiency, coagulation disorder, fungal rhinosinusitis, eosinophilic granulomatous polyangiitis, or cystic fibrosis. Single cell suspension was obtained from 10 tonsil patients including 3 who had concurrent autoimmune diseases (autoimmune disease, SLE, and psoriatic arthritis) and 8 NP patients were performed flow cytometry and ELISpot. Samples from another 7 tonsillar patients and 5 CRSwNP patients were performed cell

sorting followed by ELISpot. Finally, samples from 5 tonsillar patients and 5 CRSwNP patients were used for scRNA-seq and CITE-seq.

Tissue collection and cell isolation

Nasal polyp and tonsil tissue were collected at the time of surgery and immediately placed in PBS-Tween supplemented with a cocktail of protease inhibitors (Sigma-Aldrich, St Louis, Mo) at a 1:100 dilution. The tissue was placed in RPMI (Corning, NY) with 10% fetal bovine serum (ThermoFisher, Waltham, MA) and 1% penicillin-streptomycin. Tissue was first fragmented and then treated with 30 µg/ml DNase I and 1 mg/ml collagenase at 37°C for 30 minutes. Then further processed using a gentleMACS dissociator (Miltenyi Biotec, San Diego, CA), strained through a 70 µm mesh, and red blood cells were eliminated using an EasySep™ RBC Depletion kit. Then, the cells were ready for single cell suspensions for flow cytometry and sorting or 10xgenomics studies as described below. Cell viability was determined by trypan blue staining using a Cellometer Auto X4 automated cell counter.

Flow cytometry and sorting

Cells were first stained with Aqua LIVE/DEAD staining reagent (Invitrogen, CA, USA) to discriminate between live and dead cells. Cells were incubated with an Fc block reagent (Miltenyi Biotec, CA, USA) at 4ºC for 10 min in the dark. Antibody staining was performed for 30 min at 4ºC in the dark: APC-Cy7 mouse anti-human CD19 (BD, SJ25C1, 0.2mg/ml), Alexa700 mouse anti-human CD3 (Invitrogen, UCHT1, 0.1mg/ml), PE mouse anti-human CD27 (BD, MT271, 0.0125mg/ml), PerCP-Cy5.5 mouse anti-human CD38 (BD, HIT2, 0.2mg/ml), FITC mouse anti-human IgD (BD, IA6-2, 0.0125mg/ml), GPR183 (Biolegend, SA313E4,

0.2mg/ml). Flow cytometry was performed on an LSRII (BD Biosciences, NJ) and sorting was performed on FACSAria II at the Flow Cytometry Core Facility at Northwestern University. Compensation beads (BD biosciences) were used for single stained controls, and fluorescence minus one (FMO) control was used as negative controls to determine gates. All analysis and compensation were performed with FlowJo software (BD Biosciences).

ELISpot

ELISpot assay was performed using kits from MabTech, according to the manufacturer's instructions. Dispersed single cells were resuspended in RPMI with 10% FCS and 1% penicillin-streptomycin and added in triplicate on the hydrophobic high-protein-binding immobilon 96-well ELISpot plates (MAIPS4510).

Plates were pre-coated overnight with either dsDNA or capturing anti-IgG. Then wells were washed thoroughly with 1xPBS containing 0.1% Tween 20. Ten-fold serial dilutions were used to determine a suitable starting cell numbers (10^3 cells for IgG and 10^5 cells for dsDNA IgG ASCs), followed by incubation at 37°C for 24 hours. Next, biotinylated anti-IgG was added to the plate and incubated for 2 hours, followed by streptavidin conjugated horseradish peroxidase (HRP) incubation for 1 hour. Then, TMB substrate was filtered through a 0.45 μm filter and 100 μl/well was added and incubated for 5 minutes at RT. The reaction was stopped by dH_2O, and then left the plate to completely dry, and analyzed using a CTL-Immunospot S6 analyzer (Cellular Technologies Ltd). Only the clear and darker spots correspond to the footprint of individual ASCs, and the smaller and paler spots were considered as background due to nonspecific binding. Wells used as blank controls were incubated with PBS only or cells only without coating.

Human monoclonal 3H9 antibody, which is specifically against dsDNA, ssDNA, nucleosomes, and cardiolipin was used as a positive control for anti-dsDNA-specific binding (2, 3). HEK293A cells were transfected with a total of 9 μg 3H9 plasmids (generously provided by Dr. Patrick Wilson from University of Chicago, 1:1 ratio of kappa and heavy chain-plasmids were used) using PEI or Lipofectamine 3000 Transfection Reagent (Invitrogen) following the manufacturer's instructions. The transfected cells were incubated at 37°C for 48 hours, then washed with 1X PBS before performing ELISpot.

10xgenomics scRNA-seq and CITE-seq

scRNA-seq: Samples with >80% cell viability were used library preparation.10x Genomics scRNA-seq 3' mRNA and cell surface protein libraries were generated according to the manufacturer's instructions. Single-cell libraries (mRNA and antibody-derived tags (ADT) were generated using the Chromium Controller Single-Cell instrument and Chromium Single Cell 3' Library & Gel Bead kit v3. The mRNA and ADT libraries were combined at a 4:1 ratio and sequenced with an Illumina NovaSeq 6000 at Northwestern University Next Generation Sequencing Core facilities. Raw sequencing data were converted to demultiplexed fastq files using Cell Ranger pipeline (mkfastq and count), and gene counts were computed by aligning to the human genome GRCh38 and then analyzed using R4.4.1 with Seurat toolkit package 4.2.0 (4). We utilized the Seurat package in R to analyze the unique molecular identifier (UMI)-collapsed cell-by-gene matrix. Each sample (5 NPs and 5 tonsils) was initially processed as an individual matrix, and then merged, log normalized, findVariableFeatures, scaled, and centered. Cells with mitochondrial gene expression over 15%, ribosome gene expression over 30%, or

UMI counts outside of 200-20000 were removed. To avoid biased clustering, we regressed out the number of UMIs in each cell and the percentage of mitochondrial and ribosomal transcripts.

A list of the 2000 most variable genes was generated using FindVariableFeatures function with selection method = “vst”. Principal component analysis (PCA) was performed over this list of variable genes and first 15 principal components were selected for further analysis based on elbow plot of percentage of variance explained per principal components.

A shared nearest neighbor (SNN) graph and uniform manifold approximation and projection embedding (UMAP) were constructed on the first 15 PCs, then FindClusters was used to determine clusters, which were graphically displayed using UMAP with a resolution of 0.2. For differential gene expression of B lineage cells, FindMarkers function was used to compare the transcriptomic difference between donors with a minimum of 10% of all cells, a minimum of 0.25 average log fold-change, and a maximum of 0.05 adjusted p-value. Additional analysis of B lineage cells was conducted to compare the transcriptomic difference between donors using FindMarkers, and comparisons between specific genes were visualized by violin and feature plots.

CITE-seq (Cellular Indexing of Transcriptomes and Epitopes by sequencing**)**: Antibody labeling followed the manufacturer’s instructions: cells were labeled with TotalSeqB antibodies (Biolegend): CD19 (HIB19, 0.5mg/ml), CD27 (O323, 0.5mg/ml), CD38 (HIT2, 0.5mg/ml), IgD (IA6-2, 0.5mg/ml), phycoerythrin (PE)-anti-human GPR183 (SA313E4, 0.1mg/ml), and anti-PE (PE001, 0.5mg/ml) following the manufacturer’s instructions. 1.5 million live cells from each sample were resuspended in the staining buffer with human TruStain Fc blocking reagent and labeled with TotalSeqB antibody pool using 1μg of each antibody. Followed by the computational pipeline discussed above, the demultiplexed ADT reads were processed and

matched with their corresponding ADT sequences and assigned to cell barcodes. Cells with ADT counts outside of the range of 300-3000 were excluded from the analysis. This step is in parallel with the cell removal with the criteria based on RNA levels.

GSEA and GO analysis

GSEA (Gene set enrichment analysis): performed on the top 2000 most highly variable features based on normalized raw counts in ASCs derived from NPs and tonsils. GO analysis was performed on genes with log fold change >1 in the two group comparisons.

RESULTS

NPs contained increased anti-dsDNA IgG and total IgG ASCs

A total of 10^5 cells for dsDNA-specific and 10^3 cells for total- IgG were deposited on each ELISpot well. Uncoated wells with cells or coated wells with no cells were plated as controls. To assess the dsDNA specific ELISpot assay, positive controls were performed by using human 3H9 antibody transfected HEK293A cells and an expected high frequency of dsDNA-specific ASCs were observed (Fig. E2). The frequencies of IgG ASCs among total B cells between tonsils, autoimmune-tonsils, and NPs was compared. The frequencies of IgG ASC per B cell as well as dsDNA autoreactive ASCs per B cell were higher in NPs than tonsils and autoimmune-tonsils (Figs. E3A, B). Further, the ratio of dsDNA autoreactive ASCs per total ASCs was also subsequently higher in NPs (1.5%, n=8), and this abundance was comparable to those in autoimmune-tonsils (1.8%, n=3, $p<0.001$) (Fig. E3C), indicating a potential role of autoantibodies in CRSwNP pathogenesis.

scRNA-seq identified transcriptionally distinct B cell subsets

Since negative selection kits for separating B cells from tissue are not available, epithelial cells (characteristic genes in parentheses) including basal (*KRT5*), apical (*KRT8*), and glandular (*LYZ*), as well as ciliated cells (*PIFO*), fibroblasts (*LUM*), endothelial cells (*SPARCL1*), mast cells (*TPSAB1*), club cells (*PIGR*), and a small number of T cells (*IL32*) were also identified. The characteristic genes for each cluster were visualized by feature plots (Figs. E3B, E4). These results demonstrate that scRNA-seq could separate B cell subsets, especially ASCs using the single cell transcriptome.

REFERENCE

E1. Meltzer EO, Hamilos DL, Hadley JA, Lanza DC, Marple BF, Nicklas RA, et al. Rhinosinusitis: Establishing definitions for clinical research and patient care. Otolaryngol Head Neck Surg. 2004;131(6 Suppl):S1-62.

E2. Shlomchik MJ, Aucoin AH, Pisetsky DS, Weigert MG. Structure and function of anti-DNA autoantibodies derived from a single autoimmune mouse. Proc Natl Acad Sci U S A. 1987;84(24):9150-4.

E3. Andrews SF, Huang Y, Kaur K, Popova LI, Ho IY, Pauli NT, et al. Immune history profoundly affects broadly protective B cell responses to influenza. Sci Transl Med. 2015;7(316):316ra192.

E4. Butler A, Hoffman P, Smibert P, Papalexi E, Satija R. Integrating single-cell transcriptomic data across different conditions, technologies, and species. Nature Biotechnology. 2018;36(5):411-20.

Figure E1

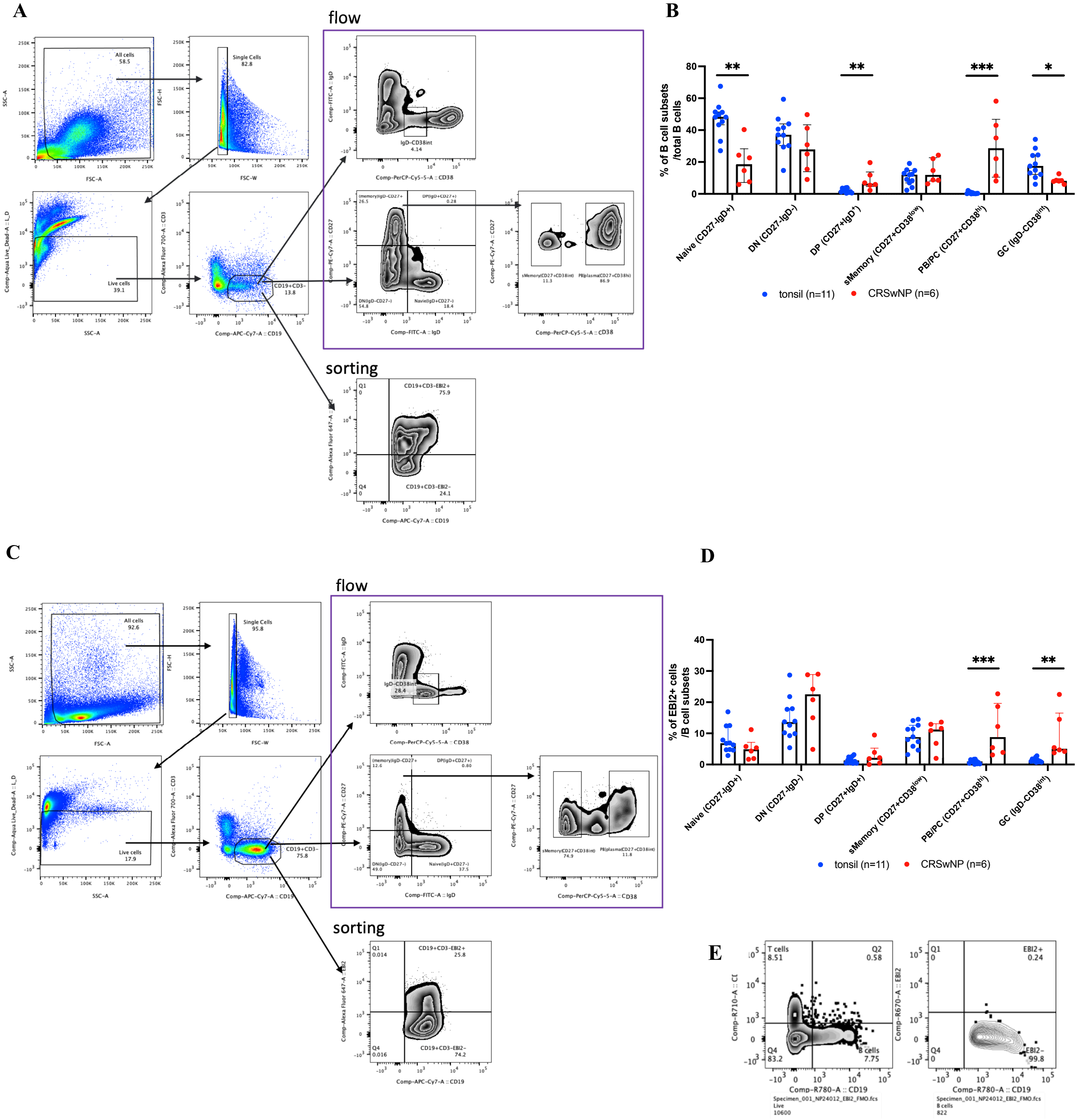

Figure E1. A. Representative phenotype of B cell subsets in NPs as assessed using flow cytometry. All cells were identified after gating on single alive cells. Total B cell (CD3-CD19+) frequency was calculated from the CD3- gate. B lineage cells include naïve (IgD+CD27-),

memory (IgD-CD27+), GC (IgD-CD38^{int}), and plasmablasts (CD27+CD38^{hi}) were gated on B cells. **B**. Bar plot of frequency of naïve, memory, DP, DN, GC, PB, and PC are expressed as a % of total CD19+ cells. The sorting was gated on EBI2 and CD19. **C**. Representative phenotype of B cell subsets in tonsils as assessed using flow cytometry. **D**. Bar plot of frequency of B subsets expressing EBI2 was calculated on CD19+EBI2+ gate. **E**. Gating strategy for EBI2 using fluorescence minus one (FMO) control.

Figure E2

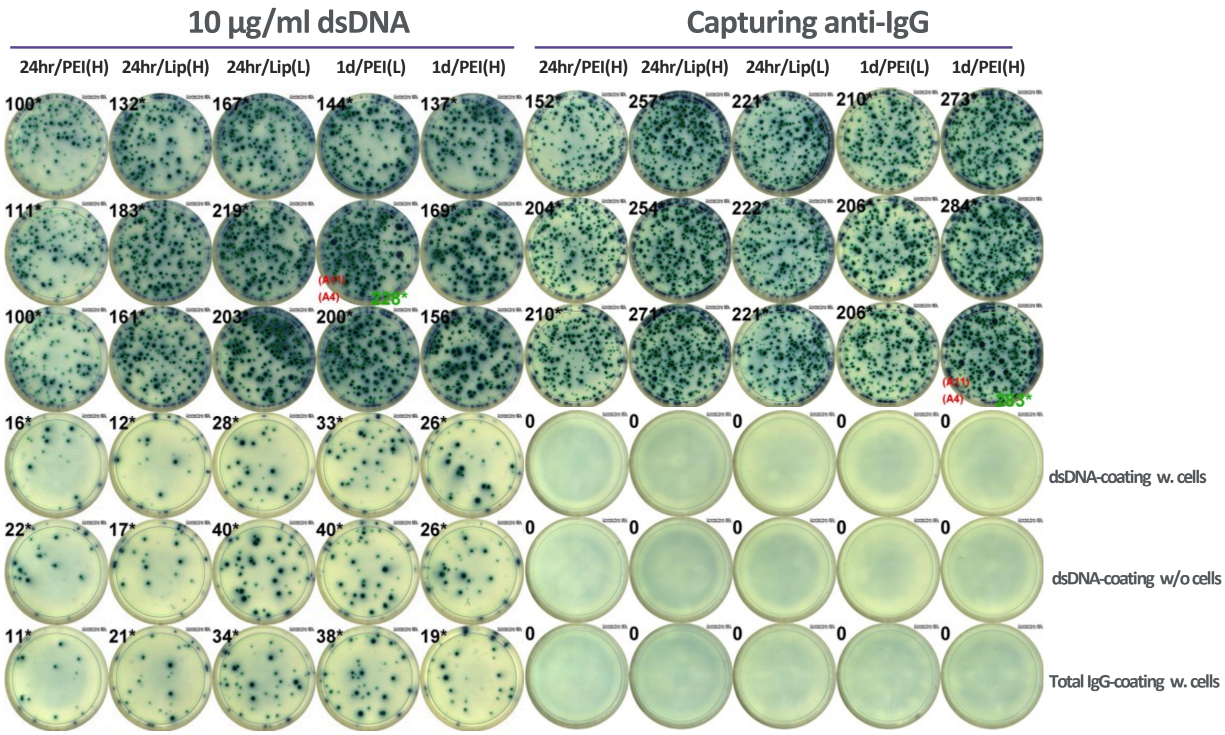

Figure E2. Validating dsDNA-IgG specific and total IgG ELISpot assays using HEK293A transfected 3H9 mutant. HEK293A cells transfected with 9μg 3H9 heavy and light chain plasmids (a kind gift from Dr. Patrick Wilson lab, University of Chicago, IL), specific monoclonal antibody specifically against dsDNA. 10 μg/ml dsDNA or capturing anti-IgG were pre-coated the microplates. PEI2-transfected protocol and lipofectamine 3000 (Invitrogen) were transfected and compared their transformation efficiency. Pre-coating with dsDNA or total IgG with or without non-transfected cells was used as negative control.

Figure E3

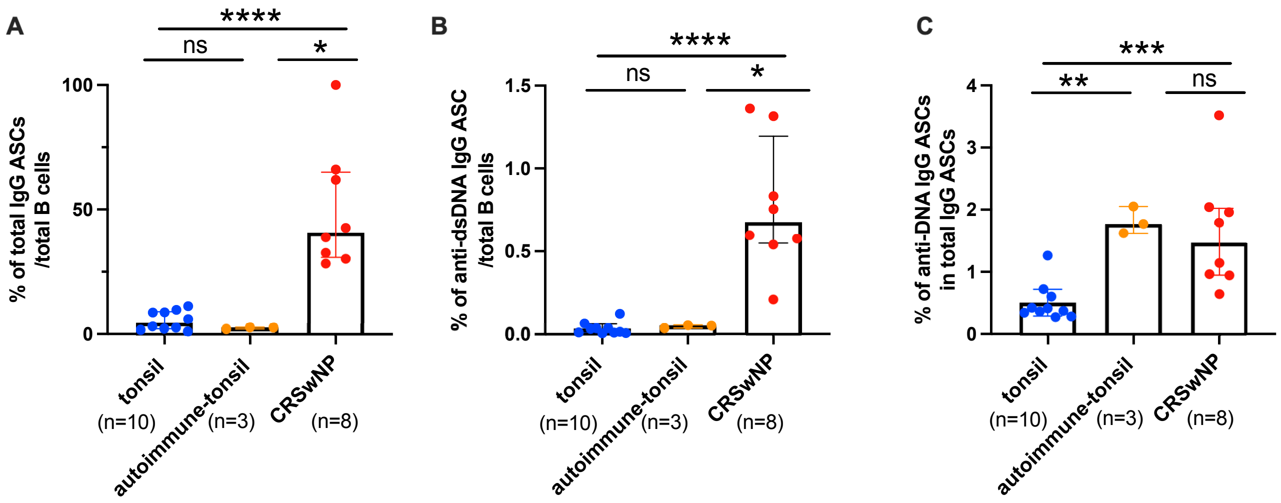

Figure E3: Comparisons of frequencies of ASCs between tonsils, autoimmune tonsils, and NPs. The frequencies of IgG ASCs in total B cells (**A**), anti-dsDNA IgG ASCs in total B cells (**B**), and anti-dsDNA IgG ASCs in total IgG ASCs (**C**).

Figure E4

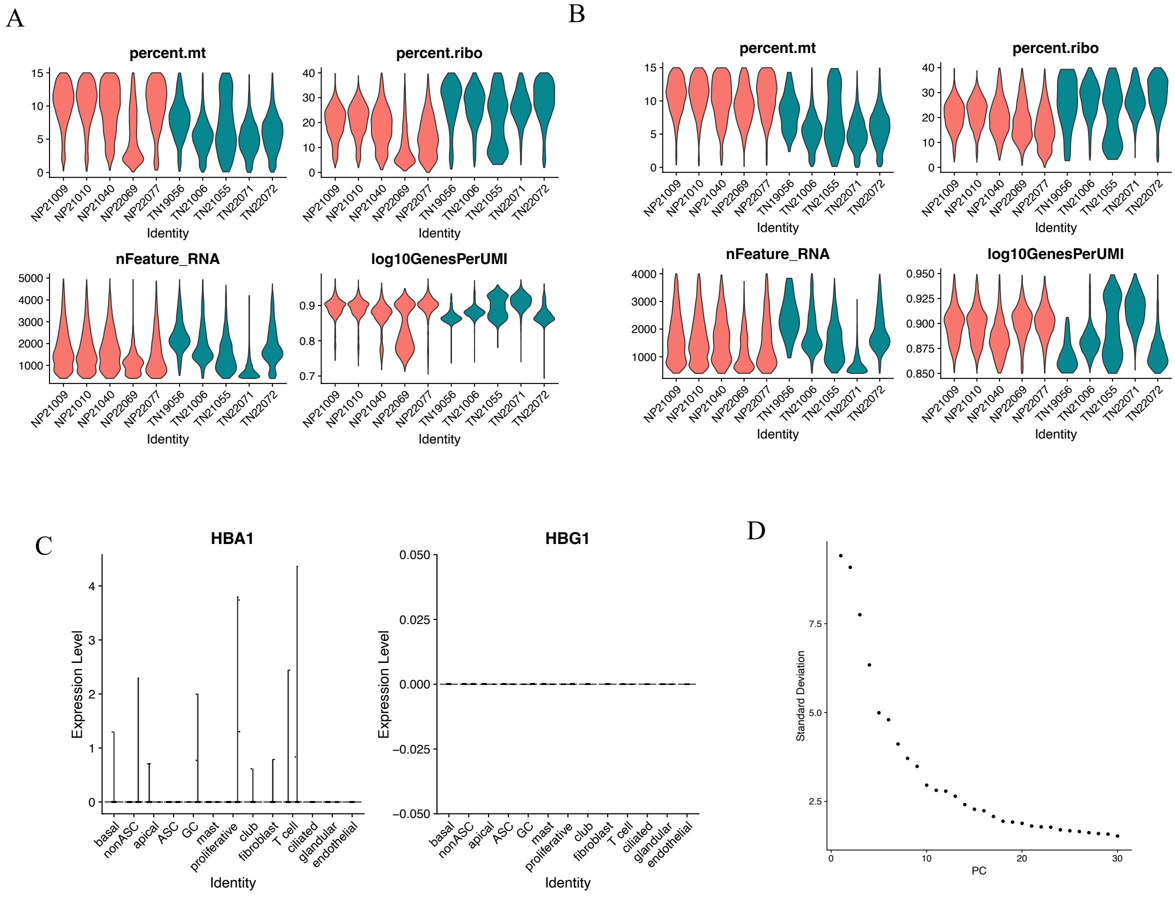

Figure E4: Quality control of scRNA-seq. Violin plot of quality metrics including mitochondrial percentages, ribosomal gene percentage, total genes, and complexity shown by donor prior (**A**) and post removing of low-quality cells (**B**). Violin plot of hemoglobin genes HBA1 and HBG1 distributed by cell type (**C**). Elbow plot was used to determine the top 15 of 30 principal components (PCs) used for visualization by UMAP (**D**).

Figure E5:

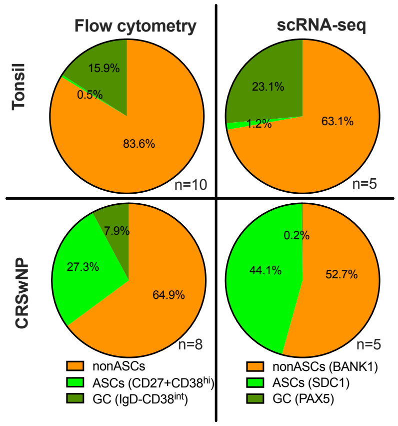

Figure E5: Pie chart of frequencies of B cell subpopulations. Pie chart of frequencies of B cell subpopulations including nonASCs, ASCs, and GC cells resulted from flow cytometry on the left side and scRNA-seq on the right side.

Figure E6:

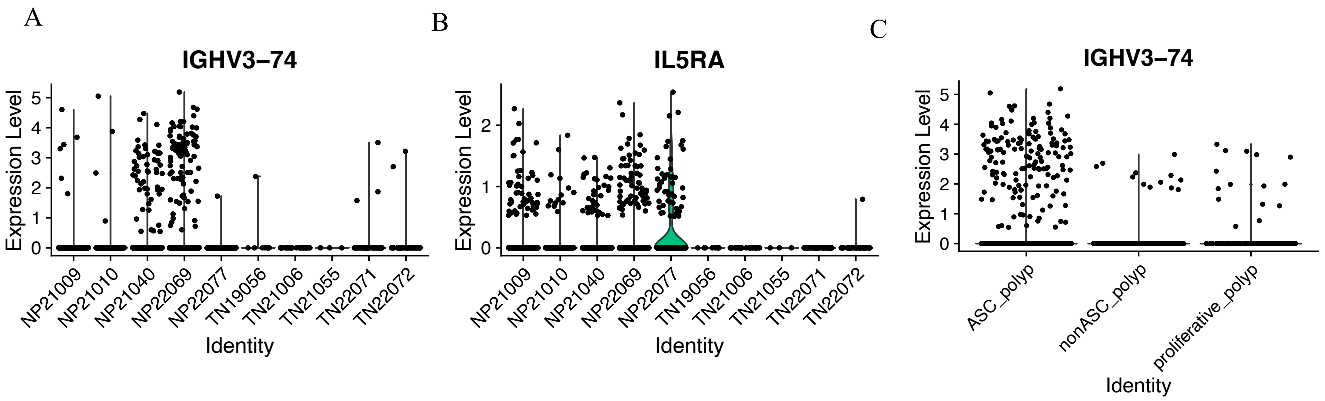

Figure E6: Violin plots of IGHV3-74 and IL5RA. Violin plots showing gene expression across single cells from individual donors for IGHV3-74 (A), IL5RA (B), and the expression of IGHV3-74 in each of the identified B cell subsets in NPs**.**

Table E1. Upregulated genes in CRSwNP

Gene	p_val	avg_log2FC	pct.1 (ASC-polyp)	pct.2 (ASC-tonsil)	p_val_adj	FC (NP/tonsil)
IGHA2	7.78E-16	3.80990	0.53400	0.18300	0.00000	14.0
IGHE	1.60E-08	3.35767	0.12700	0.00700	0.00048	10.3
IGHG4	3.84E-09	3.04931	0.85800	0.60600	0.00012	8.3
CCND2	1.88E-21	2.21187	0.54700	0.09900	0.00000	4.6
IGHV3-74	1.31E-32	2.11275	0.19200	0.02100	0.00000	4.3
JCHAIN	7.30E-38	2.10271	0.97800	0.85900	0.00000	4.3
IGHA1	3.61E-26	1.96760	0.85700	0.54200	0.00000	3.9
TXNIP	1.34E-16	1.40625	0.69700	0.32400	0.00000	2.7
SLPI	8.34E-17	1.28834	0.35800	0.00000	0.00000	2.4
IGKV1-5	0.00016685	1.18979	0.21200	0.07700	1.00000	2.3
RRBP1	3.90E-14	1.15687	0.88000	0.60600	0.00000	2.2
CITED2	2.71E-12	1.13110	0.58000	0.23900	0.00000	2.2
HIST1H2BG	4.18E-10	1.10721	0.48200	0.19000	0.00001	2.2
SDC1	3.20E-16	1.08954	0.51500	0.13400	0.00000	2.1
H1FX	9.64E-14	1.08451	0.66400	0.29600	0.00000	2.1
FOSB	3.58E-09	1.08127	0.62900	0.35900	0.00011	2.1
SERPINB3	1.05E-12	1.02943	0.29200	0.00700	0.00000	2.0
IGKV4-1	0.07147864	0.99193	0.13300	0.07700	1.00000	2.0
TFF3	7.97E-12	0.98323	0.26300	0.00000	0.00000	2.0
S100A6	2.63E-12	0.95646	0.50700	0.18300	0.00000	1.9
BPIFB1	3.64E-10	0.89775	0.22800	0.00000	0.00001	1.9
HIST1H1D	5.82E-07	0.88657	0.45600	0.21100	0.01745	1.8
PTP4A3	9.90E-09	0.87212	0.24400	0.02800	0.00030	1.8
PECAM1	2.17E-13	0.86915	0.40800	0.07700	0.00000	1.8
ITGA6	1.26E-08	0.85058	0.35700	0.11300	0.00038	1.8
NEAT1	1.13E-11	0.85040	0.95800	0.69000	0.00000	1.8
JSRP1	5.02E-09	0.84930	0.36000	0.11300	0.00015	1.8
KRT19	7.86E-10	0.84425	0.22100	0.00000	0.00002	1.8
CST1	1.67E-10	0.84133	0.23500	0.00000	0.00001	1.8
PDK1	2.20E-11	0.84076	0.57300	0.23900	0.00000	1.8
AC103591.3	7.90E-06	0.83445	0.34400	0.15500	0.23691	1.8
NFKBIA	7.80E-08	0.83389	0.35800	0.12000	0.00234	1.8
WFDC2	6.70E-09	0.83097	0.21300	0.00700	0.00020	1.8
GAS6	3.16E-07	0.83049	0.30100	0.09200	0.00947	1.8
AC253572.2	7.28E-08	0.82947	0.33500	0.09900	0.00219	1.8
GPR183	3.57E-34	0.82042	0.35400	0.13800	0.00000	1.8

XBP1	8.73E-09	0.80575	0.87000	0.14200	0.00026	1.7
PRDM1	6.23E-11	0.76202	0.69000	0.12700	0.02100	1.7
FNDC3B	1.05E-09	0.74083	0.52200	0.21100	0.00003	1.7
AHNAK	5.50E-08	0.73182	0.31600	0.08500	0.00165	1.7
CD63	8.00E-09	0.73120	0.72700	0.44400	0.00024	1.7
IER2	3.07E-05	0.73115	0.64500	0.45100	0.92256	1.7
IGLV2-11	0.00139706	0.70722	0.15200	0.04900	1.00000	1.6
DUSP5	5.27E-12	0.70718	0.00700	0.10600	0.00000	1.6
ATF5	9.56E-06	0.70389	0.26300	0.09200	0.28692	1.6
JUN	3.03E-06	0.70192	0.82200	0.56300	0.09104	1.6
IGLV2-14	0.01577285	0.69336	0.14800	0.07000	1.00000	1.6
SLC38A2	7.06E-05	0.69329	0.51900	0.32400	1.00000	1.6
ANXA1	5.57E-07	0.68473	0.15500	0.00000	0.01673	1.6
SOCS3	0.00031647	0.68277	0.23200	0.09900	1.00000	1.6
CDKN1A	0.00081365	0.67759	0.17400	0.06300	1.00000	1.6
TIMP1	5.57E-07	0.67571	0.34200	0.12000	0.01672	1.6
ELL2	3.55E-08	0.67563	0.53200	0.26100	0.00107	1.6
IL5RA	8.21E-08	0.66808	0.18700	0.00700	0.00246	1.6
POSTN	1.11E-06	0.66501	0.14800	0.00000	0.03340	1.6
SCGB1A1	1.03E-07	0.65240	0.17300	0.00000	0.00309	1.6
EMP3	2.14E-05	0.65175	0.46100	0.26800	0.64170	1.6
FOXO3	1.92E-05	0.65061	0.26800	0.10600	0.57511	1.6
TMEM107	3.55E-05	0.64298	0.36800	0.19700	1.00000	1.6
UAP1	8.66E-05	0.64173	0.29200	0.14100	1.00000	1.6
IRF4	3.16E-06	0.62473	0.32800	0.31900	0.00785	1.5
AC007952.4	8.42E-08	0.60069	0.59600	0.28200	0.00253	1.5
DUSP1	7.54E-09	0.59959	0.57300	0.26100	0.00023	1.5
DDIT4	0.00842266	0.59828	0.22400	0.12700	1.00000	1.5
AC245014.3	4.58E-05	0.59742	0.25300	0.09200	1.00000	1.5
PABPC4	5.15E-05	0.59628	0.54800	0.36600	1.00000	1.5
CCR10	0.00013801	0.59414	0.19400	0.06300	1.00000	1.5
LGALS1	1.27E-05	0.58278	0.54800	0.31700	0.38218	1.5
TXNDC5	1.71E-05	0.58194	0.86700	0.73200	0.51417	1.5
CASP10	0.00014675	0.57863	0.24700	0.10600	1.00000	1.5
IGKC	0.00014985	0.57542	1.00000	0.93700	1.00000	1.5
TPM4	0.00410404	0.57403	0.46600	0.34500	1.00000	1.5
SEL1L	3.76E-07	0.57310	0.69700	0.42300	0.01128	1.5
SPAG4	6.85E-06	0.57150	0.31100	0.12000	0.20547	1.5
CAPN2	0.00026027	0.57122	0.17400	0.05600	1.00000	1.5

MAPKAPK2	0.00069341	0.57078	0.30000	0.16200	1.00000	1.5
SLC7A5	0.00023207	0.57025	0.21300	0.07700	1.00000	1.5
AQP3	0.00134501	0.56706	0.24600	0.12700	1.00000	1.5
CCR2	8.50E-06	0.56462	0.12600	0.00000	0.25513	1.5
ARRDC3	0.00028474	0.56431	0.27000	0.12700	1.00000	1.5
ATF3	0.00199282	0.56417	0.10100	0.02100	1.00000	1.5
TTC3	0.00136998	0.55893	0.53500	0.35900	1.00000	1.5
CEP128	0.02781663	0.55869	0.17300	0.09900	1.00000	1.5
C12orf57	0.00039630	0.55706	0.51500	0.35200	1.00000	1.5
CREB3L2	0.00016605	0.55513	0.47100	0.29600	1.00000	1.5
TMEM238	1.10E-05	0.55320	0.22500	0.06300	0.33151	1.5
HIPK2	5.90E-05	0.55276	0.30300	0.13400	1.00000	1.5
IER3	3.63E-05	0.55275	0.13700	0.01400	1.00000	1.5
TRIB1	0.00010099	0.55212	0.47800	0.28200	1.00000	1.5
SNHG5	0.00010466	0.55046	0.35800	0.19000	1.00000	1.5
TENT5C	0.00012931	0.55032	0.72100	0.50000	1.00000	1.5
ALDH1L2	0.00048650	0.55003	0.20500	0.07700	1.00000	1.5
DENND3	0.00026963	0.54858	0.14600	0.03500	1.00000	1.5
CRYBG3	0.00030854	0.54546	0.12300	0.02100	1.00000	1.5
TNFAIP3	0.00037356	0.54456	0.16700	0.04900	1.00000	1.5
KIF21A	1.74E-05	0.53987	0.13000	0.00700	0.52311	1.5
CAV1	0.00011986	0.53960	0.19400	0.06300	1.00000	1.5
TNFAIP2	0.00180830	0.53896	0.11400	0.02800	1.00000	1.5
ALOX15	3.19E-05	0.53726	0.11100	0.00000	0.95846	1.5
TXN	5.15E-05	0.53703	0.37900	0.20400	1.00000	1.5
ITM2C	5.41E-05	0.53697	0.64500	0.43700	1.00000	1.5
KDELR1	7.42E-05	0.53688	0.56400	0.36600	1.00000	1.5
ITGA8	0.00011360	0.53672	0.11100	0.00700	1.00000	1.5
ELF3	7.96E-05	0.53670	0.10100	0.00000	1.00000	1.5
MIAT	0.00025049	0.53616	0.11400	0.01400	1.00000	1.5
FCRL5	6.33E-05	0.53609	0.64900	0.45100	1.00000	1.5
FXYD3	6.99E-05	0.51997	0.10200	0.00000	1.00000	1.4
HIST1H2BF	0.03495529	0.50912	0.12100	0.06300	1.00000	1.4
AGR2	1.89E-05	0.49645	0.11700	0.00000	0.56612	1.4
FNDC3A	5.45E-06	0.48556	0.52600	0.28200	0.16343	1.4
JUNB	3.09E-05	0.48551	0.55800	0.33800	0.92635	1.4
CHST15	0.02427451	0.47872	0.15900	0.09200	1.00000	1.4
NDRG1	0.00097724	0.47431	0.11100	0.02100	1.00000	1.4
RPN1	0.00045809	0.47004	0.51800	0.35900	1.00000	1.4

CCDC88C	0.00765681	0.46838	0.21800	0.12700	1.00000	1.4
ERN1	0.01655413	0.46827	0.30700	0.21100	1.00000	1.4
PIM2	1.19E-24	0.46299	0.68500	0.59600	0.00000	1.4
ATXN1	0.00653945	0.44769	0.24700	0.14800	1.00000	1.4
SCARB2	0.00053796	0.44506	0.22700	0.09900	1.00000	1.4
HIST1H2AC	0.03873963	0.44310	0.29700	0.21100	1.00000	1.4
IGF1	8.49E-05	0.44236	0.12700	0.01400	1.00000	1.4
DDX3Y	3.64E-05	0.40847	0.11000	0.00000	1.00000	1.3
CLCC1	0.01554969	0.40356	0.19600	0.11300	1.00000	1.3
CCNL2	0.12930195	0.40341	0.17000	0.12700	1.00000	1.3
EGR1	0.00206429	0.40334	0.21900	0.10600	1.00000	1.3
GBP5	0.02424656	0.39870	0.13500	0.07000	1.00000	1.3
ABCA5	0.00053406	0.39009	0.18300	0.06300	1.00000	1.3
LMNA	0.02207791	0.36770	0.18700	0.11300	1.00000	1.3
HSP90B1	3.27E-17	0.36615	0.99100	0.97300	0.00000	1.3
FTL	0.07364281	0.35763	0.91100	0.91500	1.00000	1.3
IRF2BP2	0.01109390	0.30740	0.22100	0.13400	1.00000	1.2
IGLC2	0.49423595	0.28268	0.81100	0.90100	1.00000	1.2
PLD3	0.01577839	0.28085	0.26000	0.16200	1.00000	1.2
MLXIP	0.03247429	0.27938	0.18000	0.11300	1.00000	1.2
PERP	4.73E-05	0.27542	0.10700	0.00000	1.00000	1.2
PRDX4	7.34E-05	0.27287	0.74400	0.57700	1.00000	1.2
MAGED1	0.03172374	0.27210	0.17000	0.10600	1.00000	1.2
ACAT1	0.01095744	0.26852	0.12000	0.04900	1.00000	1.2
AC012181.1	0.00371832	0.26494	0.12600	0.04200	1.00000	1.2
RSRC1	0.00662641	0.26439	0.20500	0.10600	1.00000	1.2
UGGT2	0.00560937	0.26341	0.10800	0.03500	1.00000	1.2
RDX	0.00168432	0.26309	0.23000	0.11300	1.00000	1.2
ITGA4	0.00011869	0.25836	0.20500	0.06300	1.00000	1.2
AC010168.2	0.02122312	0.25806	0.11000	0.04900	1.00000	1.2
PHPT1	0.00021695	0.25568	0.50000	0.30300	1.00000	1.2
GARS	6.58E-05	0.25466	0.25400	0.09900	1.00000	1.2
PHIP	0.02447028	0.25344	0.29400	0.19700	1.00000	1.2
DPEP1	0.00463940	0.25230	0.12600	0.04200	1.00000	1.2
CD59	0.00033220	0.25120	0.34900	0.19700	1.00000	1.2
LIME1	0.01756339	0.25082	0.46300	0.36600	1.00000	1.2
ACTN4	0.08541455	0.24017	0.19200	0.14100	1.00000	1.2

Table E2. Upregulated genes in tonsils

Gene	p_val	avg_log2FC	pct.1 (ASC-polyp)	pct.2 (ASC-tonsil)	p_val_adj	FC (tonsil/NP)
HLA-DRA	3.5102E-80	-2.827517	0.094	0.782	1.053E-75	7.1
HLA-DRB1	1.7366E-62	-2.815451	0.12	0.711	5.211E-58	7.0
IGHGP	3.1165E-12	-2.765064	0.124	0.359	9.351E-08	6.8
CD79A	3.9719E-44	-2.395048	0.7	0.923	1.192E-39	5.3
IGHM	5.2237E-23	-2.271536	0.218	0.627	1.567E-18	4.8
HLA-DPA1	1.2597E-48	-2.228034	0.102	0.592	3.780E-44	4.7
CD74	4.9118E-33	-2.206133	0.665	0.923	1.474E-28	4.6
HLA-DRB5	1.5588E-85	-2.169455	0	0.514	4.677E-81	4.5
VPREB3	4.3532E-59	-2.114337	0.035	0.507	1.306E-54	4.3
HLA-DQA1	5.6566E-56	-2.018349	0.032	0.479	1.697E-51	4.1
IGLC3	3.85E-11	-1.977602	0.661	0.569	1.150E-06	3.9
TMSB10	1.9258E-28	-1.959841	0.652	0.859	5.778E-24	3.9
RGS13	4.895E-56	-1.934745	0.009	0.387	1.469E-51	3.8
LAPTM5	3.0226E-38	-1.825840	0.091	0.507	9.070E-34	3.5
HLA-DPB1	1.5336E-52	-1.796013	0.054	0.528	4.602E-48	3.5
MS4A1	3.452E-58	-1.744205	0.025	0.479	1.036E-53	3.4
HLA-DQB1	5.3308E-36	-1.656715	0.069	0.451	1.600E-31	3.2
RASSF6	3.0904E-37	-1.626292	0.054	0.415	9.273E-33	3.1
CCDC144A	7.1473E-13	-1.617147	0.133	0.359	2.145E-08	3.1
TMSB4X	1.0044E-27	-1.616867	0.635	0.915	3.014E-23	3.1
RGS2	1.16E-11	-1.605268	0.146	0.352	3.663E-07	3.0
ACTB	5.9155E-17	-1.590278	0.557	0.81	1.775E-12	3.0
HSP90AA1	5.9861E-05	-1.472026	0.681	0.683	1.000E+00	2.8
IGHG2	1.7047E-05	-1.463490	0.213	0.373	5.115E-01	2.8
CD22	7.9749E-40	-1.411388	0.015	0.317	2.393E-35	2.7
STK17B	9.6053E-12	-1.398628	0.249	0.472	2.882E-07	2.6
HLA-DMA	8.0437E-28	-1.362010	0.145	0.514	2.414E-23	2.6
ARPC3	2.1007E-10	-1.313322	0.374	0.563	6.303E-06	2.5
HSPA1A	0.00465699	-1.279537	0.124	0.204	1.000E+00	2.4
DNAJB1	0.00782126	-1.260781	0.133	0.211	1.000E+00	2.4
PLCG2	0.0016417	-1.255892	0.317	0.408	1.000E+00	2.4
SERF2	5.2109E-18	-1.253579	0.781	0.866	1.564E-13	2.4
ACTG1	2.151E-15	-1.244278	0.529	0.782	6.454E-11	2.4
SNHG29	7.6599E-09	-1.240113	0.294	0.479	2.298E-04	2.4
ISG20	3.3967E-09	-1.236356	0.586	0.697	1.019E-04	2.4
HLA-A	7.7258E-19	-1.227037	0.632	0.845	2.318E-14	2.3

SYNE2	0.00185985	-1.188647	0.346	0.415	1.000E+00	2.3
SH3BGRL3	3.4034E-13	-1.170715	0.275	0.535	1.021E-08	2.3
POLD4	9.5746E-15	-1.170167	0.251	0.514	2.873E-10	2.3
HLA-C	2.1726E-22	-1.169751	0.832	0.88	6.519E-18	2.2
ATP5MG	4.3593E-08	-1.164742	0.611	0.725	1.308E-03	2.2
CD37	7.2139E-18	-1.145930	0.101	0.373	2.165E-13	2.2
H3F3A	3.3509E-10	-1.145498	0.687	0.761	1.005E-05	2.2
CCDC88A	7.0669E-07	-1.142862	0.319	0.472	2.121E-02	2.2
CD79B	1.1298E-15	-1.130380	0.235	0.507	3.390E-11	2.2
HSP90AB1	5.4825E-06	-1.119636	0.573	0.676	1.645E-01	2.2
CD52	7.5784E-12	-1.105048	0.104	0.317	2.274E-07	2.2
ZNF331	0.01143499	-1.099385	0.079	0.141	1.000E+00	2.1
CLEC2D	4.0929E-22	-1.098029	0.051	0.303	1.228E-17	2.1
GAPDH	9.5565E-11	-1.090695	0.586	0.725	2.868E-06	2.1
RGS1	1.2464E-05	-1.079197	0.342	0.465	3.740E-01	2.1
MARCKSL1	4.6178E-20	-1.062153	0.034	0.246	1.386E-15	2.1
GSTP1	2.1958E-08	-1.057786	0.693	0.754	6.589E-04	2.1
PFN1	4.2405E-11	-1.056399	0.314	0.549	1.272E-06	2.1
UBA52	3.4089E-16	-1.051329	0.722	0.887	1.023E-11	2.1
FTH1	1.0297E-12	-1.041107	0.74	0.838	3.090E-08	2.1
PRDX1	4.7731E-10	-1.033794	0.58	0.697	1.432E-05	2.0
SEPTIN6	1.7218E-07	-1.023010	0.216	0.394	5.166E-03	2.0
ARPC1B	7.6085E-10	-1.020845	0.145	0.338	2.283E-05	2.0
PARP1	8.9504E-10	-1.016333	0.393	0.592	2.686E-05	2.0
CFL1	1.8831E-07	-1.016145	0.351	0.521	5.650E-03	2.0
HLA-B	3.0731E-14	-1.015073	0.871	0.901	9.221E-10	2.0
MZB1	3.987E-16	-1.009683	0.943	0.951	1.196E-11	2.0
CD53	5.0739E-15	-0.997526	0.171	0.444	1.522E-10	2.0
LTB	8.8099E-26	-0.991600	0.009	0.204	2.643E-21	2.0
YWHAB	9.8097E-10	-0.988690	0.313	0.528	2.944E-05	2.0
SEPTIN7	1.3557E-08	-0.982146	0.291	0.479	4.068E-04	2.0
LRMP	2.6491E-14	-0.974217	0.107	0.345	7.949E-10	2.0
FAM107B	1.076E-08	-0.970562	0.235	0.423	3.229E-04	2.0
JUND	2.9034E-05	-0.965338	0.778	0.782	8.712E-01	2.0
PTPRC/CD45	3.8573E-11	-0.957721	0.227	0.458	1.157E-06	1.9
BASP1	1.2732E-16	-0.940975	0.091	0.338	3.820E-12	1.9
GAS5	3.9993E-06	-0.933039	0.237	0.387	1.200E-01	1.9
MYL6	7.0541E-06	-0.932406	0.678	0.732	2.117E-01	1.9
ARHGDIB	7.0042E-08	-0.928577	0.244	0.423	2.102E-03	1.9

CYTIP	1.0345E-07	-0.925982	0.354	0.507	3.104E-03	1.9
OST4	2.8754E-07	-0.919846	0.614	0.683	8.628E-03	1.9
PDIA4	4.7319E-08	-0.916994	0.573	0.669	1.420E-03	1.9
TCEA1	7.0391E-07	-0.914418	0.414	0.535	2.112E-02	1.9
PPIB	2.195E-07	-0.906908	0.791	0.817	6.586E-03	1.9
FAU	8.0901E-16	-0.900667	0.865	0.944	2.428E-11	1.9
OAZ1	3.8909E-13	-0.898221	0.728	0.831	1.168E-08	1.9
HNRNPA1	3.1583E-06	-0.894451	0.351	0.507	9.477E-02	1.9
ST6GAL1	7.1498E-08	-0.893437	0.357	0.521	2.145E-03	1.9
RABAC1	4.8431E-06	-0.880659	0.694	0.754	1.453E-01	1.8
PTMA	6.6558E-11	-0.878864	0.901	0.908	1.997E-06	1.8
PDIA3	0.00019812	-0.873569	0.621	0.62	1.000E+00	1.8
NCF1	3.7657E-08	-0.868071	0.24	0.43	1.130E-03	1.8
ARPC5	1.7159E-05	-0.867294	0.228	0.373	5.149E-01	1.8
RFTN1	1.0605E-24	-0.865878	0.022	0.246	3.182E-20	1.8
CLIC1	7.7666E-08	-0.863825	0.319	0.493	2.330E-03	1.8
BTG1	2.8553E-05	-0.863603	0.516	0.627	8.568E-01	1.8
SNHG8	0.00019624	-0.863349	0.247	0.366	1.000E+00	1.8
TNFRSF13C	1.3997E-10	-0.861150	0.096	0.289	4.200E-06	1.8
HSPA1B	0.29623232	-0.859765	0.13	0.155	1.000E+00	1.8
RACK1	9.2762E-10	-0.847523	0.787	0.81	2.783E-05	1.8
MYDGF	1.6592E-06	-0.842385	0.572	0.655	4.978E-02	1.8
SERP1	7.1156E-09	-0.841499	0.74	0.796	2.135E-04	1.8
TMBIM6	3.4629E-10	-0.840171	0.665	0.768	1.039E-05	1.8
TMEM123	1.1465E-06	-0.830048	0.137	0.289	3.440E-02	1.8
NPM1	3.8345E-06	-0.829530	0.453	0.577	1.151E-01	1.8
EZR	0.00119826	-0.827363	0.341	0.423	1.000E+00	1.8
BTF3	7.5047E-05	-0.825372	0.45	0.521	1.000E+00	1.8
ARPC2	9.9239E-07	-0.824554	0.512	0.627	2.978E-02	1.8
CXCR4	9.8486E-05	-0.819468	0.158	0.282	1.000E+00	1.8
CAP1	2.7626E-07	-0.814259	0.215	0.387	8.290E-03	1.8
CHCHD2	1.2848E-05	-0.804111	0.569	0.641	3.855E-01	1.7
CCM2	1.052E-09	-0.797278	0.174	0.387	3.157E-05	1.7
EEF1A1	2.5695E-11	-0.797066	0.977	0.979	7.710E-07	1.7
CD83	1.139E-14	-0.796126	0.016	0.155	3.418E-10	1.7
IGHD	3.1616E-20	-0.796011	0.042	0.275	9.487E-16	1.7
IGHG1	1.5201E-10	-0.795299	0.732	0.481	4.561E-06	1.7
LY9	8.7192E-08	-0.791855	0.11	0.275	2.616E-03	1.7
PCED1B-AS1	1.0661E-08	-0.789436	0.266	0.472	3.199E-04	1.7

ATP5MF	4.9697E-06	-0.788611	0.341	0.493	1.491E-01	1.7
PHACTR1	3.4545E-06	-0.788164	0.08	0.204	1.037E-01	1.7
ERH	0.0001044	-0.780664	0.249	0.38	1.000E+00	1.7
EIF1	6.2788E-10	-0.778570	0.885	0.93	1.884E-05	1.7
CD38	0.08801323	-0.771745	0.468	0.43	1.000E+00	1.7
B2M	1.5348E-18	-0.768467	0.971	0.993	4.605E-14	1.7
IGHG3	1.6074E-11	-0.768464	0.738	0.431	4.823E-07	1.7
VDAC1	0.00047197	-0.766629	0.2	0.303	1.000E+00	1.7
TMED9	0.00010242	-0.765240	0.49	0.577	1.000E+00	1.7
SEC61B	8.1608E-09	-0.760100	0.73	0.81	2.449E-04	1.7
SET	3.5494E-05	-0.759152	0.379	0.493	1.000E+00	1.7
EVI2B	0.00258761	-0.758925	0.319	0.415	1.000E+00	1.7
HCST	8.5682E-06	-0.757331	0.108	0.239	2.571E-01	1.7
MYL12A	8.599E-08	-0.752655	0.249	0.451	2.580E-03	1.7
LINC01480	7.432E-06	-0.747178	0.083	0.204	2.230E-01	1.7
GNG5	2.0333E-05	-0.746327	0.322	0.458	6.101E-01	1.7
UQCR10	0.00107179	-0.744446	0.349	0.444	1.000E+00	1.7
FAM3C	0.001936	-0.742944	0.257	0.345	1.000E+00	1.7
NSMCE3	3.8748E-06	-0.742911	0.067	0.183	1.163E-01	1.7
NDUFB2	0.00023237	-0.739474	0.273	0.394	1.000E+00	1.7
SELENOH	2.6028E-06	-0.739453	0.292	0.458	7.810E-02	1.7
COX6B1	0.00104442	-0.735382	0.466	0.521	1.000E+00	1.7
LCP1	8.2821E-10	-0.733884	0.096	0.282	2.485E-05	1.7
MEF2C	0.0003231	-0.733251	0.585	0.592	1.000E+00	1.7
GNAI2	7.7144E-08	-0.725437	0.225	0.415	2.315E-03	1.7
ELK3	4.6143E-07	-0.725058	0.158	0.324	1.385E-02	1.7
COX7C	2.9056E-05	-0.723497	0.57	0.641	8.719E-01	1.7
PDIA6	2.705E-07	-0.720470	0.655	0.739	8.117E-03	1.6
CD27	0.00106205	-0.718891	0.459	0.542	1.000E+00	1.6
TPD52	3.86E-06	-0.717670	0.431	0.556	1.158E-01	1.6
UBE2D2	0.00223754	-0.717594	0.257	0.345	1.000E+00	1.6
IFI27L2	3.4889E-05	-0.714260	0.251	0.394	1.000E+00	1.6
HMGB1	9.869E-08	-0.713627	0.699	0.803	2.961E-03	1.6
COPE	2.1757E-06	-0.713609	0.56	0.669	6.528E-02	1.6
CORO1A	3.9659E-09	-0.712866	0.135	0.331	1.190E-04	1.6
NDUFA4	0.00040684	-0.709562	0.564	0.606	1.000E+00	1.6
ETS1	2.0801E-12	-0.708690	0.061	0.246	6.242E-08	1.6
LSM7	1.9843E-07	-0.708629	0.24	0.43	5.954E-03	1.6
EML6	1.3226E-07	-0.707992	0.056	0.183	3.969E-03	1.6

SSR2	0.00081386	-0.703607	0.618	0.62	1.000E+00	1.6
TMEM243	1.4737E-05	-0.701954	0.129	0.254	4.422E-01	1.6
HLA-E	1.7123E-08	-0.701828	0.643	0.725	5.138E-04	1.6
TRMT112	0.00024477	-0.700416	0.387	0.486	1.000E+00	1.6
ATP5MC3	0.05157035	-0.699999	0.408	0.444	1.000E+00	1.6
NDUFA1	2.4809E-05	-0.698792	0.496	0.577	7.444E-01	1.6
LIMD2	1.9297E-08	-0.698598	0.105	0.275	5.790E-04	1.6
PKM	5.2888E-06	-0.693013	0.151	0.303	1.587E-01	1.6
TMA7	8.5899E-07	-0.692575	0.463	0.592	2.577E-02	1.6
PSMB9	1.12E-08	-0.691828	0.19	0.38	3.361E-04	1.6
TERF2IP	4.7905E-05	-0.689545	0.234	0.366	1.000E+00	1.6
ITPR2	0.00010236	-0.687128	0.171	0.296	1.000E+00	1.6
PTP4A2	1.6079E-05	-0.686701	0.303	0.451	4.825E-01	1.6
JAK1	0.00038397	-0.685597	0.281	0.394	1.000E+00	1.6
POU2F2	2.617E-06	-0.684901	0.273	0.444	7.853E-02	1.6
C4orf3	0.00032584	-0.683294	0.303	0.415	1.000E+00	1.6
IGHV3-30	0.00153315	-0.680370	0.041	0.106	1.000E+00	1.6
NANS	0.00024006	-0.678988	0.279	0.387	1.000E+00	1.6
HSBP1	0.00159501	-0.666345	0.167	0.268	1.000E+00	1.6
HMCES	1.6284E-07	-0.665187	0.114	0.275	4.886E-03	1.6
EDF1	0.01070934	-0.663751	0.548	0.57	1.000E+00	1.6
TOMM7	1.3755E-05	-0.663170	0.469	0.577	4.127E-01	1.6
TPT1	1.293E-07	-0.661908	0.972	0.979	3.880E-03	1.6
REL	1.4223E-15	-0.661810	0.016	0.162	4.268E-11	1.6
HSPE1	0.01976001	-0.657745	0.304	0.366	1.000E+00	1.6
CALR	0.00057015	-0.655376	0.721	0.704	1.000E+00	1.6
NDUFB4	0.0375994	-0.655018	0.396	0.408	1.000E+00	1.6
SRP19	0.00358423	-0.654276	0.351	0.43	1.000E+00	1.6
SRP14	0.00147659	-0.654267	0.522	0.57	1.000E+00	1.6
P4HB	1.65E-05	-0.653086	0.509	0.62	4.951E-01	1.6
SHOC2	2.1142E-07	-0.652662	0.083	0.225	6.344E-03	1.6
MZT2B	0.02547697	-0.652126	0.465	0.5	1.000E+00	1.6
RNGTT	3.9047E-08	-0.651285	0.085	0.239	1.172E-03	1.6
HLA-DOB	6.0727E-15	-0.648836	0.085	0.317	1.822E-10	1.6
RAB11FIP1	0.00019283	-0.648589	0.095	0.197	1.000E+00	1.6
SUMO2	0.00333735	-0.647992	0.351	0.437	1.000E+00	1.6
AC114760.2	7.8494E-14	-0.644624	0.038	0.211	2.355E-09	1.6
COMMD6	0.00264122	-0.644373	0.249	0.338	1.000E+00	1.6
ZNF706	0.00032094	-0.644086	0.437	0.521	1.000E+00	1.6

LSM8	3.9705E-06	-0.641546	0.216	0.38	1.191E-01	1.6
HCLS1	0.0012833	-0.640781	0.199	0.303	1.000E+00	1.6
LRPAP1	0.00171926	-0.638076	0.349	0.451	1.000E+00	1.6
TPI1	0.00058858	-0.633938	0.322	0.444	1.000E+00	1.6
IGHG3	1.6074E-11	-0.632664	0.738	0.831	4.823E-07	1.6
ACADM	5.4236E-05	-0.632603	0.127	0.254	1.000E+00	1.6
CCNI	0.01064377	-0.632376	0.469	0.486	1.000E+00	1.6
SWAP70	1.2792E-14	-0.630156	0.031	0.197	3.838E-10	1.5
ELF1	0.00120825	-0.630100	0.181	0.289	1.000E+00	1.5
MOB1A	3.136E-05	-0.629991	0.139	0.268	9.410E-01	1.5
STK17A	4.3295E-11	-0.629822	0.047	0.204	1.299E-06	1.5
PPIA	0.01295293	-0.628215	0.456	0.514	1.000E+00	1.5
MRPS24	0.00111121	-0.623706	0.348	0.444	1.000E+00	1.5
TCL1A	7.6465E-23	-0.622587	0.001	0.148	2.294E-18	1.5
TIMM17A	0.01054062	-0.622472	0.164	0.239	1.000E+00	1.5
SEM1	0.00357993	-0.620984	0.263	0.359	1.000E+00	1.5
CUTA	0.00505708	-0.619360	0.491	0.535	1.000E+00	1.5
CD46	0.00101192	-0.619203	0.272	0.387	1.000E+00	1.5
DAD1	0.00141097	-0.617152	0.537	0.577	1.000E+00	1.5
JPX	7.2657E-06	-0.614879	0.173	0.331	2.180E-01	1.5
CTSH	5.2717E-06	-0.614226	0.221	0.387	1.582E-01	1.5
LMAN2	0.00069296	-0.613299	0.423	0.521	1.000E+00	1.5
HMGN2	0.01636486	-0.613171	0.307	0.387	1.000E+00	1.5
COX4I1	0.00340546	-0.612931	0.566	0.599	1.000E+00	1.5
NACA	0.00041092	-0.611135	0.788	0.803	1.000E+00	1.5
NDUFB8	0.00397181	-0.611133	0.323	0.43	1.000E+00	1.5
GLRX	0.00664103	-0.608413	0.348	0.408	1.000E+00	1.5
NAP1L1	0.00198329	-0.605411	0.276	0.366	1.000E+00	1.5
RHOA	0.00124822	-0.604645	0.291	0.394	1.000E+00	1.5
YWHAZ	0.01588905	-0.602244	0.36	0.408	1.000E+00	1.5
ARPC4	2.6825E-09	-0.599580	0.095	0.275	8.049E-05	1.5
BACH1	0.00172643	-0.599406	0.175	0.282	1.000E+00	1.5
HSPD1	0.03795742	-0.597654	0.341	0.394	1.000E+00	1.5

1. Meltzer EO, Hamilos DL, Hadley JA, Lanza DC, Marple BF, Nicklas RA, et al. Rhinosinusitis: Establishing definitions for clinical research and patient care. Otolaryngol Head Neck Surg. 2004;131(6 Suppl):S1-62.
2. Shlomchik MJ, Aucoin AH, Pisetsky DS, Weigert MG. Structure and function of anti-DNA autoantibodies derived from a single autoimmune mouse. Proc Natl Acad Sci U S A. 1987;84(24):9150-4.
3. Andrews SF, Huang Y, Kaur K, Popova LI, Ho IY, Pauli NT, et al. Immune history profoundly affects broadly protective B cell responses to influenza. Sci Transl Med. 2015;7(316):316ra192.
4. Butler A, Hoffman P, Smibert P, Papalexi E, Satija R. Integrating single-cell transcriptomic data across different conditions, technologies, and species. Nature Biotechnology. 2018;36(5):411-20.
